# UDiTaS™, a genome editing detection method for indels and genome rearrangements

**DOI:** 10.1186/s12864-018-4561-9

**Published:** 2018-03-21

**Authors:** Georgia Giannoukos, Dawn M. Ciulla, Eugenio Marco, Hayat S. Abdulkerim, Luis A. Barrera, Anne Bothmer, Vidya Dhanapal, Sebastian W. Gloskowski, Hariharan Jayaram, Morgan L. Maeder, Maxwell N. Skor, Tongyao Wang, Vic E. Myer, Christopher J. Wilson

**Affiliations:** 1 Editas Medicine, 11 Hurley Street, Cambridge, MA 02141 USA; 2 Present Address: Arrakis Therapeutics, 35 Gatehouse Drive, Waltham, MA 02451 USA

**Keywords:** Gene editing, CRISPR/CAS9, Next generation sequencing, NGS, UDiTaS, Translocation detection

## Abstract

**Background:**

Understanding the diversity of repair outcomes after introducing a genomic cut is essential for realizing the therapeutic potential of genomic editing technologies. Targeted PCR amplification combined with Next Generation Sequencing (NGS) or enzymatic digestion, while broadly used in the genome editing field, has critical limitations for detecting and quantifying structural variants such as large deletions (greater than approximately 100 base pairs), inversions, and translocations.

**Results:**

To overcome these limitations, we have developed a Uni-Directional Targeted Sequencing methodology, UDiTaS, that is quantitative, removes biases associated with variable-length PCR amplification, and can measure structural changes in addition to small insertion and deletion events (indels), all in a single reaction. We have applied UDiTaS to a variety of samples, including those treated with a clinically relevant pair of *S. aureus* Cas9 single guide RNAs (sgRNAs) targeting *CEP290*, and a pair of *S. pyogenes* Cas9 sgRNAs at T-cell relevant loci. In both cases, we have simultaneously measured small and large edits, including inversions and translocations, exemplifying UDiTaS as a valuable tool for the analysis of genome editing outcomes.

**Conclusions:**

UDiTaS is a robust and streamlined sequencing method useful for measuring small indels as well as structural rearrangements, like translocations, in a single reaction. UDiTaS is especially useful for pre-clinical and clinical application of gene editing to measure on- and off-target editing, large and small.

**Electronic supplementary material:**

The online version of this article (10.1186/s12864-018-4561-9) contains supplementary material, which is available to authorized users.

## Background

Common assays for genome editing involve PCR amplification of a targeted genomic region and subsequent analysis, either by endonuclease cleavage at base mismatches [1, 2] or sequencing [3–5]. However, PCR-mediated assays are fundamentally unable to measure structural changes to the genome in conjunction with small indels. Unintended translocations and other structural changes have been specifically called out for investigation in genome editing therapies by the NIH recombinant DNA advisory Committee (RAC) [6] and the FDA [7]. Measuring structural changes has recently become more feasible using a method called AMP-Seq (Anchored Multiplex PCR sequencing) [8] that is intended for clinical detection of oncogenic translocations and a similar method called HTGTS (High throughput, genome-wide translocation sequencing) [9] or LAM-HTGTS (linear amplification-mediated-HTGTS) [10]. GUIDE-seq [11], a modification of AMP-seq, is a powerful tool to capture de novo off-target editing by CRISPR RNA-guided nucleases. All these methods utilize a target specific primer in addition to an adapter ligated universal priming site on sheared DNA to achieve “uni-directional” amplification, and sequencing. However, DNA shearing is a cumbersome step in the library preparation process for all these methods: it requires specialized equipment and it is not readily amenable to studies with large numbers of samples. Consequently, it has not been broadly applied in the gene editing field. In addition, shearing DNA has been shown to induce DNA damage that results in base miscalling [12, 13], something potentially problematic when trying to assess gene editing frequencies at low levels (e.g.*:* less than 1%).

**Fig. 1:**
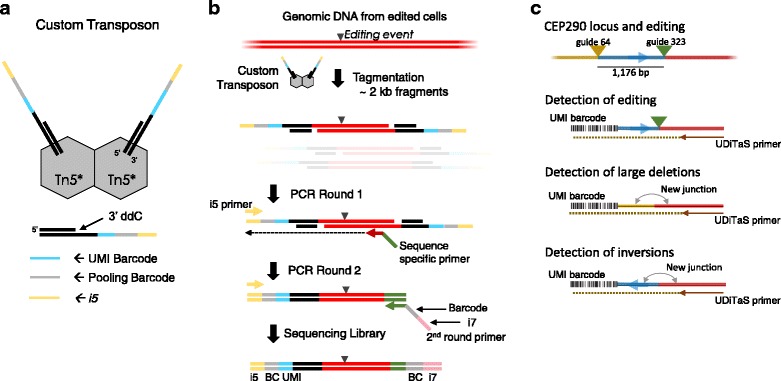
Schematics depicting key components, process, and applications for UDiTaS. A custom hyperactive Tn5 enzyme [14, 15] (Tn5*) with UMI, pooling barcode, and i5 sequence is assembled **a**. and used to tagment genomic DNA (**b**, top). Target regions are amplified with a sequence specific “anchor” primer, and further amplified in a second round to create the Illumina sequencing library **b**., bottom. UDiTaS can detect and measure many event types including: small indels, large deletions (greater than > 100 bp), and inversions; the barcode represents the Unique Molecular Index (UMI) from the tagmentation step **c**

Tagmentation has emerged as a straightforward tool that simultaneously fragments and adds adapters in a fast (~ 10 min) enzymatic reaction [14–16]. In UDiTaS, a custom-designed Tn5 transposon has been developed, which contains the full-length Illumina forward (i5) adapter, a sample barcode, and unique molecule identifier (UMI) (Fig. 1a). The full UDiTaS process (Fig. 1b) is comprised of the tagmentation step, followed by two PCR steps: round one PCR uses the target specific anchor primer, and round two adds the reverse (i7) Illumina adapter and an additional sample barcode. UDiTaS has been tested with multiple primers and genomic DNA samples and is amenable to all labs using genome editing technologies with access to NGS equipment.

## Results

As a case study for assessing complex gene editing events, a pair of *S. aureus* Cas9 (SaCas9) sgRNA were used to target a region within intron 26 of the CEP290 gene. Reduction of CEP290 expression leads to several human diseases including a blindness condition termed Leber’s Congenital Amaurosis Type 10 (LCA10) [17]. The most frequent cause of LCA10 is a rare single nucleotide variant in intron 26 that creates a splice donor, leading to an additional exon, and prematurely truncating the protein [18]. Removal of the deleterious intron 26 splice donor site is predicted to restore *CEP290* gene function. A single guide RNA pair, CEP290-64 and CEP290-323, that cuts 1176 base pairs around the splice donor has been selected from an internal screen (not shown) for further characterization.

**Fig. 2:**
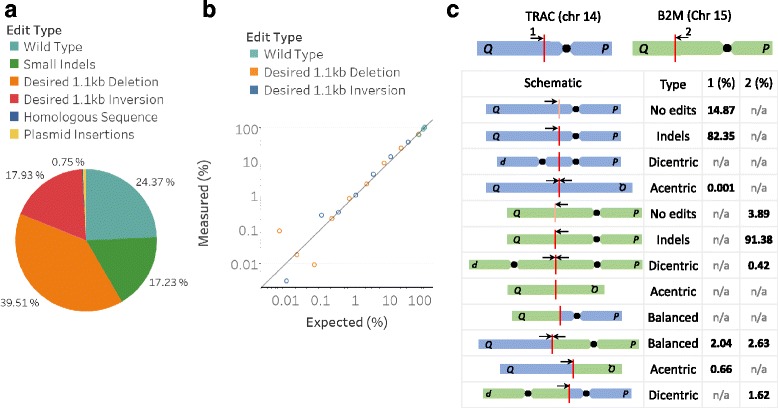
UDiTaS in practice. **a**. Editing a population of U-2 OS cells at the CEP290 IVS26 locus with two SaCas9 guides ~ 1.1 kb apart generates many possible outcomes measured and depicted in the pie chart. The events include: small indels, the desired ~ 1.1 kb deletion, the ~ 1.1 kb inversion of the intervening fragment, and homologous or sister chromosome translocations. **b**. A linearity and Lower Limit of Detection (LLoD) experiment using a clonal, engineered HEK293 cell line, mixed at various ratios with the unmodified parental line. The assay has excellent linearity, accuracy, and an LLoD of ~ 0.1%. **c**. Inter-chromosomal translocations in T-cells are measured after nucleofection with two SpCas9 RNPs, one targeting the TRAC gene and another the B2M gene. The schematic and table shows all possible outcomes and the measured result when applicable. Red lines indicate the editing sites and arrows the primers (1 = OLI6259 and 2 = OLI6256)

To illustrate the utility of UDiTaS, U-2 OS cells were transfected with linear DNA fragments expressing sgRNA CEP290-64 and CEP290-323 along with a plasmid expressing SaCas9; after three days genomic DNA was isolated, UDiTaS libraries created, sequenced on a MiSeq, and the data processed through a bioinformatics pipeline (Additional file 1 Figure S1 and Additional file 2). Using a targeted primer flanking the guide 323 cut site (Fig. 1c) a range of edits and rearrangements around the expected cut sites were observed (Additional file 3: Figure S2a-d), automatically classified, and tallied (Fig. 2a). Editing that resulted in small indel events were observed, as expected, at a rate of ~ 17%. In addition, junctions from the desired ~ 1.1 kb deletion were also present at ~ 40%. Notably, inversions of the ~ 1.1 kb fragment between the two cut sites were also observed at ~ 18%, comparable to the deletion. Other lower frequency junctions were also observed at ~ 0.75%, including translocations between homologous or sister chromosomes at the identical cut sites (Additional file 3: Figure S2 d).

To characterize the method’s linearity, accuracy, and Lower Limit of Detection (LLoD) for the desired large editing events, we constructed a stable cell line and plasmids that contained the CEP290 intron 26 wild type locus, the deletion, and the inversion. Our HEK293 cells have three copies of the CEP290 locus and the clone we created has two deletions and one inversion. Genomic DNA (gDNA) from this edited HEK293 cell line was mixed with parental, non-edited gDNA to generate a titration of the ~ 1.1 kb deletion and inversion across a five-log range. Plotting expected versus UDiTaS measured editing rates for the deletion and inversion showed excellent correlation down to approximately 0.1% (Fig. 2b). When comparing experiments on the same samples we observed high reproducibility, R^2^ = 0.99 (Additional file 4: Figure S3).

The UDiTaS protocol uses 50 ng of input DNA, which is equivalent to approximately 14,300 human haplomes. Assuming a binomial sampling distribution and ~ 20% process yield, one would expect, with 95% confidence, 2-3 observations at 0.1% in this UDiTaS library; consistent with our observations (Additional file 5: Figure S4). Increasing the sensitivity is theoretically possible by increasing the input DNA along with sequencing read depth; as both are needed to increase the number of unique UMIs in the analysis. On-target mapping rates to the genome for this study were > 95%, also indicative of the process being robust and productive (Additional file 6: Figure S5).

To further demonstrate the linearity of UDiTaS and to compare it to AMP-Seq, reference plasmids synthesized to contain the intron 26 wild type locus, large deletion, and inversion, were used as samples. These plasmids ranging from ~ 2200 to ~ 714,000 genome equivalents, were spiked into mouse genomic DNA and processed through the UDiTaS and Amp-Seq methods. UDiTaS showed excellent linearity down to the lowest dilution (Additional file 7: Figure S6a). As expected, UDiTaS and Amp-Seq yielded similar results. However, UDiTaS libraries were more linear with higher complexity given the same DNA input material (Additional file 7: Figure S6a). We attribute this to the more efficient tagmentation process compared to shearing and adapter ligation, and fewer, more streamlined processing steps that increased the overall yield. Similar results were obtained with UDiTaS when DNA was diluted without carrier mouse genomic DNA (Additional file 8: Figure S7a) further demonstrating the robustness of the process.

UDiTaS can also measure inter-chromosomal translocation rates. To demonstrate this, we simultaneously nucleofected two *S. pyogenes* CAS9 (SpCas9) ribonucleoprotein (RNP) complexes with sgRNAs targeting TRAC and B2M genes into activated human CD4^+^ T-cells. UDiTaS libraries from these cells were prepared with primers flanking each guide site. Of ten possible end joining outcomes, seven were theoretically possible to measure with the two targeting primers used in the study; and all seven of those events were detected and quantified in the experiment. These included typical NHEJ (non-homologous end joining) editing at the cut sites as well as balanced, acentric, and dicentric fusions between homologous or sister chromosomes as well as between distinct unrelated chromosomes (Fig. 2c). Although several studies have been published characterizing translocations in the context of gene editing [10, 19–21], none have holistically measured all events, relying on translocation assays that need to be pieced together and do not contextualize smaller indels rates. To our knowledge this is the first comprehensive study quantifying double gene editing translocation rates with indels. This study demonstrates inter-chromosomal translocation rates of ~ 2.5% with on-target indel editing of ~ 82% and ~ 91% (Fig. 2c) respectively for each guide RNA.

Linearity and an LLoD of ~ 0.01% of the assay at TRAC and B2M translocation loci were characterized with plasmids in a similar fashion as for the CEP290 locus (Additional file 7: Figure S6b, c and Additional file 8: Figure S7b). UDiTaS, when compared to AMP-Seq, was significantly more linear for both primers and the NGS libraries had higher complexity (unique molecules per read) further demonstrating the high sensitivity of UDiTaS to detect translocation events. Finally, because there were high single guide editing rates, we were able to cross-compare UDiTaS with methods commonly used for indel measurement and found excellent concordance with UDiTaS (Additional file 9: Figure S8).

## Discussion

We have developed a sequencing and analysis methodology that enables simultaneous measurement of small indels and larger structural rearrangements such as large deletions, inversions, and translocations. The UDiTaS method is robust, scalable, and available to any lab practicing genome editing with access to Next Generation Sequencing. Of note, this methodology may be useful in other gene editing settings. For example, multiplexing the anchor primers along with low (50 ng) input DNA will enable panels of candidate off-target editing sites to be monitored when samples are limiting. In addition, the custom transposon described here has potential to improve methods that utilize DNA shearing along with an anchor primer, such as GUIDE-Seq. We show excellent accuracy and linearity of the method for several primers. With that said, a potential limitation of the method is measurement inaccuracy that can emerge from biases of Tn5* transposition, primer binding, or amplification. As UDiTaS assays are developed, especially those with clinical implications, it is and will be important to validate and calibrate the assays with standards, as shown here using plasmids, to ensure accuracy.

## Conclusions

UDiTaS is an important new sequencing methodology for genome editing detection and analysis. It enables the accurate quantification of intended or unintended large structural changes in addition to small, more typical indels and SNVs that arise from single and dual gene edits. Detection methods, like UDiTaS, are especially important in therapeutic gene editing settings, where editing needs to be carefully monitored to assess efficacy and therapeutic risk.

## Methods

### UDiTaS and NGS methods

Detailed protocols for AMP-seq and UDiTaS are provided as Additional file 2. Modifications to the UDiTaS protocol are described in the Plasmid Sensitivity and T-cell TRAC/B2M experiments. Plasmids and Oligos are listed in Table 1 and Table 2.

**Table 1:** Plasmids used in this study

Plasmid Name (use PLAxxx)	Description
PLA380	IVT PCR template for TRAC and B2M Spy guides
PLA379	pUC57_Amp_CEP290_SNPs1
PLA370	pUC57_Amp_CEP290_large_inversion_SNPs_1
PLA367	pUC57_Amp_CEP290_large_deletion_SNPs_1
PLA371	pUC57_Amp_CEP290_large_inversion_SNPs_2
PLA368	pUC57_Amp_CEP290_large_deletion_SNPs_2
PLA372	pUC57_Amp_CEP290_large_inversion_SNPs_3
PLA369	pUC57_Amp_CEP290_large_deletion_SNPs_3
PLA377	pUC57_Amp_B2M_SNPs1
PLA378	pUC57_Amp_TRAC_SNPs1
PLA361	pUC57_Amp_B2M_TRAC5_SNPs_1
PLA362	pUC57_Amp_B2M_TRAC5_SNPs_2
PLA363	pUC57_Amp_B2M_TRAC5_SNPs_3
PLA364	pUC57_Amp_B2M_TRAC5_SNPs_4
PLA365	pUC57_Amp_B2M_TRAC5_SNPs_5
PLA366	pUC57_Amp_B2M_TRAC5_SNPs_6
PLA13	pAF003 STITCHR backbone plasmid

**Table 2:** Oligos used in this study

Oligo Name	Description	Sequence
OLI7076	Forward primer for B2M IVT	CACCGCTAGCTAATACGACTCACTATAGGCCACGGAGCGAGACATCTGTTTTAGAGCTAGAAATA
OLI7077	Forward primer for TRAC5 IVT	CACCGCTAGCTAATACGACTCACTATAGCTGGTACACGGCAGGGTCAGTTTTAGAGCTAGAAATA
OLI4610	Forward primer for TRAC5 Illuimina amplicon sequencing	ACACTCTTTCCCTACACGACGCTCTTCCGATCTGCATTTCAGGTTTCCTTGAGTGG
OLI4611	Reverse primer for TRAC5 Illuimina amplicon sequencing	GTGACTGGAGTTCAGACGTGTGCTCTTCCGATCTGCACTGTTGCTCTTGAAGTCC
OLI7078	Common reverse primer for IVT template	TTTTTTTTTTTTTTTTTTTTGCACCGACTCGGTGCCACTTTTTCAAGTTGATA
OLI6062	UDiTaS and AMP-seq gene specific primer for CEP290 guide 323	GTGACTGGAGTTCAGACGTGTGCTCTTCCGATCTGGACCATGGATGCACTCTGTAAATTCTCAT
OLI6256	UDiTaS and AMP-seq gene specific primer for B2M	GTGACTGGAGTTCAGACGTGTGCTCTTCCGATCTGCATGCCTTCTTAAACATCACGAGACTCTAA
OLI6253	UDiTaS and AMP-seq gene specific primer for TRAC5 - Forward	GTGACTGGAGTTCAGACGTGTGCTCTTCCGATCTGGTTTCTAAGATGCTATTTCCCGTATAAAGCATGA
OLI6259	UDiTaS and AMP-seq gene specific primer for TRAC5 - Reverse	GTGACTGGAGTTCAGACGTGTGCTCTTCCGATCTGCACTGTTGCTCTTGAAGTCCATAGACCTC
OLI6380	UDiTaS adapter top oligo i5_N501_UMI_Tn5-A	AATGATACGGCGACCACCGAGATCTACACTAGATCGCNNNNNNNNNNTCGTCGGCAGCGTCAGATGTGTATAAGAGACAG
OLI6381	UDiTaS adapter top oligo i5_N502_UMI_Tn5-A	AATGATACGGCGACCACCGAGATCTACACCTCTCTATNNNNNNNNNNTCGTCGGCAGCGTCAGATGTGTATAAGAGACAG
OLI6382	UDiTaS adapter top oligo i5_N503_UMI_Tn5-A	AATGATACGGCGACCACCGAGATCTACACTATCCTCTNNNNNNNNNNTCGTCGGCAGCGTCAGATGTGTATAAGAGACAG
OLI6383	UDiTaS adapter top oligo i5_N504_UMI_Tn5-A	AATGATACGGCGACCACCGAGATCTACACAGAGTAGANNNNNNNNNNTCGTCGGCAGCGTCAGATGTGTATAAGAGACAG
OLI6384	UDiTaS adapter top oligo i5_N505_UMI_Tn5-A	AATGATACGGCGACCACCGAGATCTACACGTAAGGAGNNNNNNNNNNTCGTCGGCAGCGTCAGATGTGTATAAGAGACAG
OLI6385	UDiTaS adapter top oligo i5_N506_UMI_Tn5-A	AATGATACGGCGACCACCGAGATCTACACACTGCATANNNNNNNNNNTCGTCGGCAGCGTCAGATGTGTATAAGAGACAG
OLI6386	UDiTaS adapter top oligo i5_N507_UMI_Tn5-A	AATGATACGGCGACCACCGAGATCTACACAAGGAGTANNNNNNNNNNTCGTCGGCAGCGTCAGATGTGTATAAGAGACAG
OLI6387	UDiTaS adapter top oligo	AATGATACGGCGACCACCGAGATCTACACCTAAGCCTNNNNNNNNNNTCGTCGGCAGCGTCAGATGTGTATAAGAGACAG
Tn5-A bottom	UDiTaS adapter bottom oligo	[Phos]CTGTCTCTTATACA[ddC]
OLI5589	UDiTaS and AMP-seq round 1 and 2 PCR primer P5/i5	AATGATACGGCGACCACCGAGATCTACAC
OLI5639	UDiTaS and AMP-seq round 2 PCR primer i7_N701_SBS12	CAAGCAGAAGACGGCATACGAGATAGCGGAATGTGACTGGAGTTCAGACGTGT
OLI5640	UDiTaS and AMP-seq round 2 PCR primer i7_N702_SBS12	CAAGCAGAAGACGGCATACGAGATGATCATGCGTGACTGGAGTTCAGACGTGT
OLI5641	UDiTaS and AMP-seq round 2 PCR primer i7_N703_SBS12	CAAGCAGAAGACGGCATACGAGATAAGACGGAGTGACTGGAGTTCAGACGTGT
OLI5642	UDiTaS and AMP-seq round 2 PCR primer i7_N704_SBS12	CAAGCAGAAGACGGCATACGAGATCGAGTCCTGTGACTGGAGTTCAGACGTGT
OLI5643	UDiTaS and AMP-seq round 2 PCR primer i7_N705_SBS12	CAAGCAGAAGACGGCATACGAGATTCCTCAGGGTGACTGGAGTTCAGACGTGT
OLI5644	UDiTaS and AMP-seq round 2 PCR primer i7_N706_SBS12	CAAGCAGAAGACGGCATACGAGATGTACGGATGTGACTGGAGTTCAGACGTGT
OLI5645	UDiTaS and AMP-seq round 2 PCR primer i7_N707_SBS12	CAAGCAGAAGACGGCATACGAGATCATCTCTCGTGACTGGAGTTCAGACGTGT
OLI5646	UDiTaS and AMP-seq round 2 PCR primer i7_N710_SBS12	CAAGCAGAAGACGGCATACGAGATGTCGGAGCGTGACTGGAGTTCAGACGTGT
OLI5647	UDiTaS and AMP-seq round 2 PCR primer i7_N711_SBS12	CAAGCAGAAGACGGCATACGAGATACGGAGAAGTGACTGGAGTTCAGACGTGT
OLI5648	UDiTaS and AMP-seq round 2 PCR primer i7_N712_SBS12	CAAGCAGAAGACGGCATACGAGATAGGAGATGGTGACTGGAGTTCAGACGTGT
OLI5649	UDiTaS and AMP-seq round 2 PCR primer i7_N714_SBS12	CAAGCAGAAGACGGCATACGAGATAGTACTCGGTGACTGGAGTTCAGACGTGT
OLI5650	UDiTaS and AMP-seq round 2 PCR primer i7_N715_SBS12	CAAGCAGAAGACGGCATACGAGATGGACTCTAGTGACTGGAGTTCAGACGTGT
OLI2909	Index 1 - AMP-seq top adapter	AATGATACGGCGACCACCGAGATCTACACACTGCATANNWNNWNNACACTCTTTCCCTACACGACGCTCTTCCGATC*T
OLI2910	Index 1 - AMP-seq top adapter	AATGATACGGCGACCACCGAGATCTACACAAGGAGTANNWNNWNNACACTCTTTCCCTACACGACGCTCTTCCGATC*T
Illumina forward	Round 2 barcode primer for Illumina amplicon sequencing	AATGATACGGCGACCACCGAGATCTACACNNNNNNNNACACTCTTTCCCTACACGAC
Illumina reverse	Round 2 barcode primer for Illumina amplicon sequencing	CAAGCAGAAGACGGCATACGAGATNNNNNNNNGTGACTGGAGTTCAGACGTGT
AMP-seq bottom adapter	AMP-seq bottom adapter	[Phos]GATCGGAAGAGC*C*A

### Bioinformatics pipeline

The analysis pipeline was built using python code that calls additional software for specialized steps (see Supplemental Fig. 1). Code is available at https://github.com/editasmedicine/uditas. Briefly, it consists of the following steps:

i) **Demultiplexing.** Sequencing reads were first demultiplexed into the different experiments in the run using the appropriate sequencing barcodes, allowing up to one mismatch in each barcode. UMIs for each read were extracted for further downstream analysis.
ii) **Trimming**. 3′ adapters were trimmed using cutadapt [22], version 1.9.1
iii) **Create reference amplicons**. We used the expected cut sites to build reference amplicons with the expected chromosomal rearrangements: wild type, large deletion, inversion, translocation, etc.
iv) **Alignment**. Paired reads were then globally aligned to all the reference amplicons using bowtie2 [23], version 2.1.0. Finally, samtools [24] (version 1.3-5-g664cc5f) was used to create and index sorted bam files.
v) **Alignment analysis**. Reads completely covering a window around the predicted junctions (15 bp) were extracted and the total number of unique UMIs counted.
vi) **Final genome wide analysis**. Finally, reads that could not be mapped to the reference amplicons were extracted and mapped globally using bowtie2 to the appropriate background reference genome.

### U-2 OS bulk editing transfection experiment

U-2 OS cells (ATCC) were maintained in DMEM, high glucose with Glutamax and sodium pyruvate (ThermoFisher), 10% Fetal Bovine Serum, and supplemented with 1% penicillin/streptomycin. Cells were transfected by Lonza nucleofection using the 4D nucleofector system. Briefly, 250,000 cells were transfected with 1.5μg plasmid pAF003 expressing SaCas9 driven by CMV promoter and 500 ng of linear DNA fragment expressing gRNAs driven by U6 promoter (250 ng each guide). Cells were nucleofected using the SE kit and pulse code DN-100 and plated in 6-well plates. Cells were cultured for 3 days post-nucleofection and 3 transfection technical replicates were pooled together. Genomic DNA was isolated using the Agencourt DNAdvance kit (Beckman Coulter) according to manufacturer’s instructions.

### HEK293 cell line creation at the CEP290 locus

Hek293 cells (ATCC) were maintained in DMEM, high glucose with Glutamax and sodium pyruvate (ThermoFisher), 10% Fetal Bovine Serum, and supplemented with 1% penicillin/streptomycin. Cells were transfected using the Mirus TransIT 293 kit, according to manufacturer’s instructions. Briefly, 120,000 cells were seeded in a well of a 24-well plate 24 h pre-transfection. Cells were transfected with 750 ng plasmid pAF003 expressing SaCas9 driven by CMV promoter and 250 ng of linear DNA fragment expressing gRNAs driven by U6 promoter (125 ng each guide). Following expansion, cells were trypsinized, diluted and re-plated in 96-well plates at a dilution of approximately 1 cell per every 3 wells. Cells were visually monitored to ensure single cell colonies and expanded into 24-well plates. To determine editing, genomic DNA was isolated from clones using the Agencourt DNAdvance kit (Beckman Coulter) according to manufacturer’s instructions. Clones were screened by ddPCR and verified by Sanger sequencing.

### T cell – TRAC / B2M

*Streptococcus pyogenes* guide RNAs targeting the B2M and TRAC loci were generated by in vitro transcription of a PCR product using the T7-ScribeTM Standard RNA IVT Kit (CELLSCRIPT) following the manufacturer’s protocol. The PCR product for the in vitro transcription reaction was generated using plasmid PLA380 as a template and the indicated forward primers (OLI7076 for B2M, and OLI7077 for TRAC) with a common reverse primer (OLI7078).

The in vitro transcribed guide RNAs were complexed to wild type Cas9 protein at a molar ratio of 2:1 to generate ribonucleoprotein (RNP). The complexation integrity was evaluated by differential scanning fluorimetry (DSF). In brief, 5 μL of complexed RNP was diluted in 5 μL 2X Dye Mix. The 2X Dye Mix was generated from the 5000X stock SYPRO Orange Protein Gel Stain dye (Life Technologies, S6651) in 10× HEPES-Saline solution with MgCl_2_ (Boston Bio Products, C-6767) diluted to 1X in nuclease-free water. The complexed samples and uncomplexed protein controls were placed in a 384-well plate and placed in a BioRad thermocycler using the following protocol: 1 min at 20 °C, Melt Curve from 20 °C to 95 °C with increment changes of 1 °C, 1 min at 4 °C. Successful complexation is defined as a clear temperature shift between uncomplexed control samples and complexed RNP.

Human T cells were isolated from buffy coats using Miltenyi CD4 microbeads following the manufacturer’s protocol. On day 0, T-cells were activated using Dynabeads® Human T-Activator CD3/CD28 for T Cell Expansion and Activation (ThermoFisher Scientific). Beads were removed on day 2. On day 4, cells were counted using Trypan Blue (ThermoFisher Scientific) and TC20™ Automated Cell Counter (Bio-Rad) according to manufacturer’s protocol. For each condition 500,000 T cells were resuspended in 22 μL of Primary Cell Nucleofector Solution P2 (Lonza) containing 2 μM of total RNP. Samples were transferred to 16-well NucleocuvetteTM Strips (Lonza) and electroporated using program DS130 of the 4D-NucleofectorTM System (Lonza). Cells were subsequently transferred to untreated 96-well round bottom plates and cultured in 200 μL of X-Vivo 15 media (Lonza) containing 5% Human AB Serum (Gemini BioProduct), 1.6 mg/mL N-acetylcysteine (Sigma), 2 mM L-alanyl-L-glutamine (Thermo Scientific), 50 IU/ mL IL-2 (Peprotech), 5 ng/mL IL-7 (Peprotech) and 0.5 ng/mL IL-15 (Peprotech).

Four days post nucleofection, samples were pelleted and genomic DNA purified using the Agencourt DNAdvance kit (Beckman Coulter) according to the manufacturer’s protocol.

The CRISPR targeted genomic region of TRAC was PCR amplified for subsequent Sanger sequencing using primers OLI11371 and OLI11403. Amplification was performed in a 50 μl reaction volume, consisting of 10 μL of 5X Phusion HF buffer, 0.5 μM forward primer, 0.5 μM reverse primer, 200 μM dNTP, 1.5 μL DMSO, 0.5 μl of Phusion polymerase and 25 ng of gDNA template. PCR conditions were as follows: 30 s at 98 °C for initial denaturation, followed by 40 cycles of 10s at 98 °C for denaturation, 15 s at 64 °C for annealing, 30s at 72 °C for extension, and 5 min at 72 °C for the final extension. The PCR product was purified using (1.8×) Agencourt AMPure XP beads (Beckman Coulter Agencourt AMPure XP - PCR Purification #A63882) as per the manufacturer’s protocol. The amplified locus fragments were then cloned into pCR4-TOPO vectors using the ZeroBlunt TOPO Cloning Kit (Life Technologies Zero Blunt TOPO PCR Cloning Kit for Sequencing with One Shot TOP10 Chemically Competent *E. coli* #K287540) and transformed in One Shot Top10 chemically competent *Escherichia coli* cells. Cells were plated on Carbenicillin LB agar plates and incubated overnight at 37 °C. Plasmid DNA from 96 colonies per sample was sequenced by Genewiz, Inc. using an M13 reverse primer. Analysis of indel rates was done with the Geneious Software package (Biomatters, https://www.geneious.com).

For Illumina amplicon sequencing, two rounds of amplification were performed: round 1 targets the TRAC region, and round 2 adds the full-length Illumina adapter sequence. Round 1 was performed in a 12 μl reaction volume, consisting of 6 μL of NEBNext® Ultra™ II Q5® Master Mix (New England Biolabs), 0.125 μM forward primer (OLI4610), 0.125 μM reverse primer (OLI4611), and 20 ng of gDNA template. PCR conditions were as follows: 30 s at 98 °C for initial denaturation, followed by 20 cycles of 10s at 98 °C for denaturation, 15 s at 60 °C for annealing, 30s at 72 °C for extension, and 5 min at 72 °C for the final extension. The PCR product was purified using (0.9×) Agencourt AMPure XP beads (Beckman Coulter Agencourt AMPure XP - PCR Purification #A63882) as per the manufacturer’s protocol. Round 2 was performed in a 12 μl reaction volume, consisting of 6 μL of NEBNext® Ultra™ II Q5® Master Mix (New England Biolabs), 0.5 μM forward primer (Illumina forward), 0.5 μM reverse primer (Illumina reverse), and 20 ng of gDNA template. PCR conditions were as follows: 30 s at 98 °C for initial denaturation, followed by 20 cycles of 10s at 98 °C for denaturation, 15 s at 60 °C for annealing, 30s at 72 °C for extension, and 5 min at 72 °C for the final extension. The PCR product was purified using (0.9×) Agencourt AMPure XP beads (Beckman Coulter Agencourt AMPure XP - PCR Purification #A63882) as per the manufacturer’s protocol followed by size 300-1200 bp size selection on the BluePippin (Sage Science, Beverly, MA) and loaded on the Illumina MiSeq with 10% phiX. Analysis of indel rates was done as described in Bothmer et al. [25].

UDiTaS was performed according to the detailed protocol with the following modification. After tagmentation, the enzyme was inactivated with the addition of 1 μL of 0.2% SDS, pipette mixing and 5 min room temperature incubation. The tagmented DNA was added directly into round 1 PCR using primers OLI6259 and OLI6256.

### Plasmid sensitivity experiments

For CEP290 plasmid-based sensitivity experiments PLA370, PLA367, and PLA379 were used (Additional file 8: Figure S7a). For the TRAC/B2M translocation plasmid-based sensitivity experiments PLA365, PLA366, PLA377, and PLA378 were used (Additional file 8: Figure S7b). Plasmid concentrations were determined using a NanoDrop2000 Spectrophotometer and working dilutions of 10 ng/μl were generated for all plasmids. In brief, for CEP290 sensitivity experiments the first sample consists of a 50% mix of PLA370 (Inversion) and PLA367 (Large Deletion) and contains no control plasmid (PLA379). Subsequently, 10 dilutions were generated by serially diluting the PLA370/PLA367 mix (sqrt10 dilution factor) into control plasmids (PLA379) maintaining a total plasmid concentration of 10 ng/μL throughout the different dilutions. The last sample consisted of only control plasmids (PLA379). For TRAC/B2M sensitivity experiments the first sample consists of a 50% mix of PLA365 and PLA366 and contains no control plasmids (PLA377/PLA378). Subsequently, 10 dilutions were generated by serially diluting PLA366 (sqrt10 dilution factor) into an equal mix of control plasmids PLA377/PLA378 maintaining a total plasmid concentration of 10 ng/μL throughout the different dilutions. The last sample consisted of only control plasmids (equal mix between PLA377/PLA378). All samples were subsequently subjected to UDiTaS: TRAC/B2M translocation samples were amplified with OLI6256 and OLI6259, while CEP290 plasmids were amplified with OLI6062. The UDiTaS protocol was applied with the following modifications: 6 cycles for first and second round PCR.For plasmids spiked into mouse DNA, different amounts of unique plasmids were mixed into mouse gDNA. For CEP290 plasmid spike in experiments, plasmids with the estimated copy number shown in Table 3 were spiked into 10 ng/μL mouse gDNA. For TRAC/B2M plasmid spike in experiments, plasmids were spiked into 10 ng/μL mouse gDNA as described in Table 4.

**Table 3:** Plasmids and amount used in CEP290 spike-in experiment

Plasmid Name (PLAxxx)	Expected Plasmid Copy Number (per 50 ng reaction)
PLA370	1,032,035
PLA367	326,363
PLA371	103,204
PLA368	32,636
PLA372	10,320
PLA369	3264
PLA379	4,684,365

**Table 4:** Plasmids and amount used in TRAC/B2M spike-in experiment

Plasmid Name (PLAxxx)	Expected Plasmid Copy Number (per 50 ng reaction)
PLA361	1,031,968
PLA362	326,339
PLA363	103,197
PLA364	32,634
PLA365	10,320
PLA366	3263
PLA377/PLA378 (Equal mix)	4,695,992

## Additional files

Additional file 1: **Figure S1.** Schematic of the bioinformatics pipeline for UDiTaS analysis. (PPTX 61 kb)

Additional file 2: Detailed Protocols for UDiTaS and AMP-Seq methods. (DOCX 237 kb)

Additional file 3: **Figure S2.** Example editing events. Examples of various editing events in the U-2 OS bulk editing experiment shown in the Integrated Genome Viewer (IGV) [26, 27]. A schematic on top of each view depicts the observed editing event. Reads colored in red/blue were aligned to the top/bottom genomic reference DNA sequence. Note that small indels are observed in addition to the junctions formed from the larger structural changes. These indels likely arose due to repair pathway activity prior to rearrangement. **a**. 323 site small indels. **b**. 323-64 large desired 1.1 kb deletion junction. **c**. 323-64 large desired 1.1 kb inversion junction. **d**. 323 homologous junction. (PPTX 147 kb)

Additional file 4: **Figure S3.** UDiTaS reproducibility. Identical samples were run in UDiTaS using either SDS addition or Zymo column purification after tagmentation. Measured values for the various constructs are reproducible and highly correlated across a wide range of concentrations. (PPTX 3928 kb)

Additional file 5: **Figure S4.** Binomial power calculation applied to UDiTaS. A simulated binomial distribution, plotting editing frequency (e.g.: probability of success) vs. number of unique molecular identifiers (e.g.: trials) for a given number of expected observations (1, 2, or 3). Graphs on the left are 95% confidence and right 99% confidence. (PPTX 86 kb)

Additional file 6: **Figure S5.** Genome mapping rates for UDiTaS. Individual reads map to the expected genome site with high frequency indicating the robustness of the assay. Ten distinct samples for primer OLI6062 are plotted on the x-axis and the y-axis shows the percentage or reads mapping to the expected reference amplicon for each sample. (PPTX 3767 kb)

Additional file 7: **Figure S6.** UDiTaS characterization and comparison to AMP-Seq with plasmid standards. Plasmids containing the CEP290 structural variants **a**. or the TRAC-B2M balanced translocation **b**. and **c**. were synthesized and contain engineered unique SNPs in the insert to identify the plasmid after sequencing. The plasmids were diluted at various levels into mouse genomic DNA and processed through UDiTaS and AMP-Seq using primers for CEP290 **a**., B2M **b**. and TRAC **c**. The number of input plasmids versus the number of plasmids detected is plotted for both UDiTaS and AMP-Seq. Linear regression models and 95% confidence model predictions are displayed on the plots. The parameter *β* determines the linearity of the method, with values close to 1 indicating more linearity. We used ANOVA *p*-values to examine differences in *β* for UDiTaS and AMP-Seq. Below each plot, the table displays the total number of fastq reads sequenced in the reaction, the number of reads mapped to the wild-type amplicon (the most abundant one) and the final number of UMIs counted, for both UDiTaS and AMP-Seq. At all tested loci, UDiTaS shows greater linearity and number of UMIs detected when compared to AMP-Seq. (PPTX 12722 kb)

Additional file 8: **Figure S7.** UDiTaS characterization of plasmid standards without carrier DNA. To ensure that the carrier mouse genomic DNA was not influencing the UDiTaS reaction, additional sets of UDiTaS reactions were run with plasmids in the absence of any carrier DNA. **a**. CEP290 plasmids with the Wild Type, Large Deletion, and Large Insertion (PLA379, PLA367, and PLA370) and **b**. B2M-TRAC plasmids with the B2M, TRAC, and both balanced translocations (PLA377, PLA378, PLA365, and PLA366) were diluted as described in the methods. The DNA plasmids mixtures were process through UDiTaS and the analysis pipeline. Plotted is the expected frequency for a given structural variant vs. measured frequency for a structural variant (x = y is the grey line). Accuracy and linearity appear to be excellent for both loci with all four primers, with an LLOD of ~ 0.01%-0.1%. (PPTX 991 kb)

Additional file 9: **Figure S8.** Comparison of Indel rates between UDiTaS and other methods. T-Cells edited with the TRAC + B2M guides were analyzed for indel editing at the TRAC locus using PCR-amplification followed by Sanger Sequencing or NGS, in addition to UDiTaS with two different anchor primers. Indel rates were very similar between the methods. (PPTX 81 kb)

## Abbreviations

AMP-seq: Anchored multiplex PCR sequencing
gDNA: Genomic DNA
GUIDE-seq: Genome-wide, unbiased identification of DSBs enabled by sequencing
HTGTS: High throughput, genome-wide translocation sequencing
LAM-HTGTS: Linear amplification-mediated-HTGTS
LCA10: Leber’s Congenital Amaurosis Type 10
LLoD: Lower Limit of Detection
NGS: Next Generation Sequencing
NHEJ: Non-homologous end joining
PCR: Polymerase Chain Reaction
RAC: Recombinant DNA Advisory Committee
RNP complex: Ribonucleoprotein complex
SaCas9: *S. aureus* Cas9
sgRNAs: single guide RNAs
SpCas9: S. pyogenes CAS9
UDiTaS: Unidirectional Targeted Sequencing
UMI: Unique molecule identifier
