# Supplementary material for: UDiTaS™, a genome editing detection method for indels and genome rearrangements
Source: BMC Genomics. 2018 Mar 21;19:212. doi: 10.1186/s12864-018-4561-9 (PMC5861650; doi:10.1186/s12864-018-4561-9)
Supplement: Supplementary file 1 — Figure S1. Schematic of the bioinformatics pipeline for UDiTaS analysis. (PPTX 61 kb) [file 12864_2018_4561_MOESM1_ESM.pptx]

## Slide 1
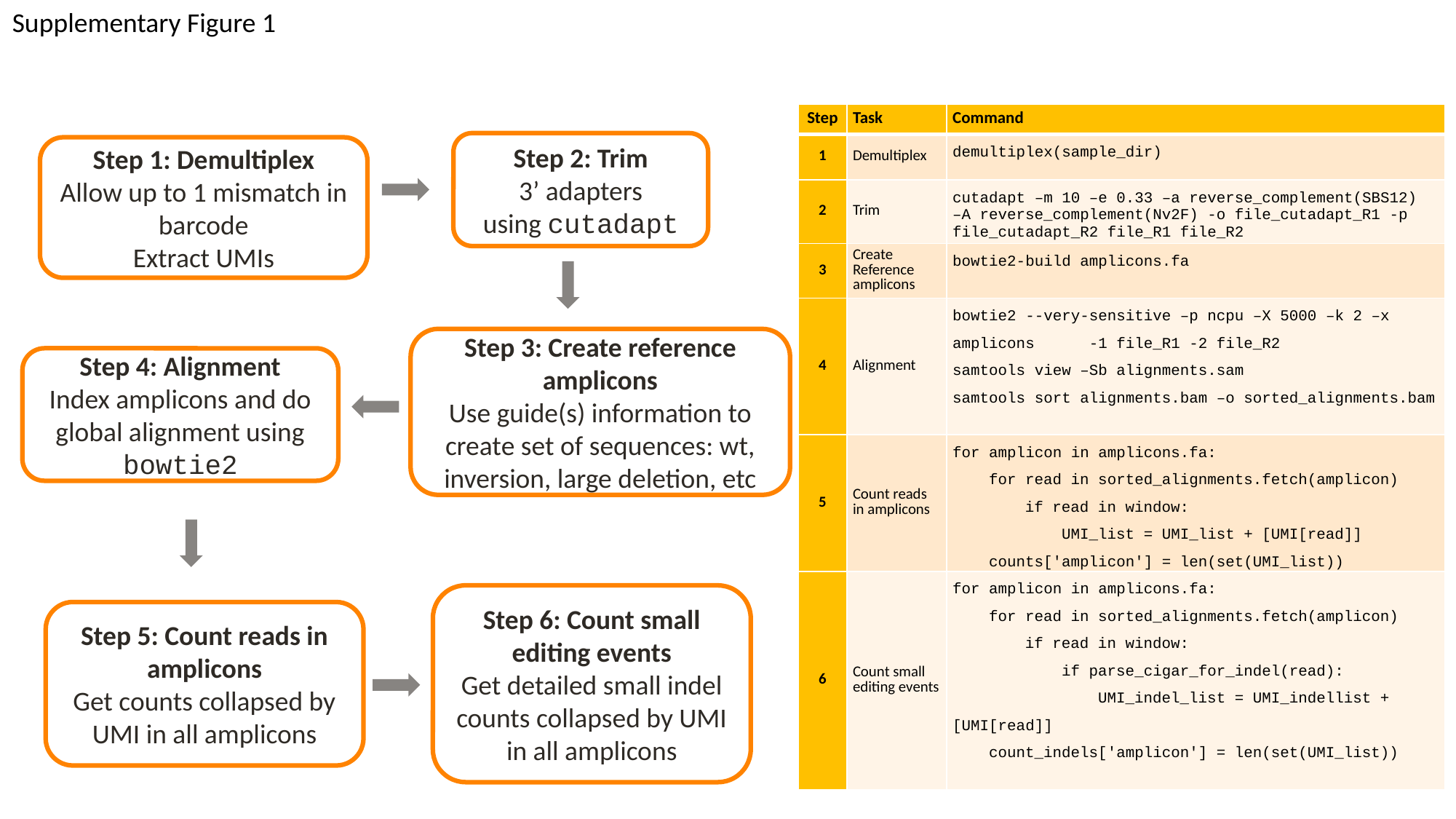

Supplementary Figure 1
| Step | Task | Command |
| --- | --- | --- |
| 1 | Demultiplex | demultiplex(sample\_dir) |
| 2 | Trim | cutadapt –m 10 –e 0.33 –a reverse\_complement(SBS12) –A reverse\_complement(Nv2F) -o file\_cutadapt\_R1 -p file\_cutadapt\_R2 file\_R1 file\_R2 |
| 3 | Create Reference amplicons | bowtie2-build amplicons.fa |
| 4 | Alignment | bowtie2 --very-sensitive –p ncpu –X 5000 –k 2 –x amplicons -1 file\_R1 -2 file\_R2 samtools view –Sb alignments.sam samtools sort alignments.bam –o sorted\_alignments.bam |
| 5 | Count reads in amplicons | for amplicon in amplicons.fa: for read in sorted\_alignments.fetch(amplicon) if read in window: UMI\_list = UMI\_list + [UMI[read]] counts['amplicon'] = len(set(UMI\_list)) |
| 6 | Count small editing events | for amplicon in amplicons.fa: for read in sorted\_alignments.fetch(amplicon) if read in window: if parse\_cigar\_for\_indel(read): UMI\_indel\_list = UMI\_indellist + [UMI[read]] count\_indels['amplicon'] = len(set(UMI\_list)) |
Step 2: Trim
3’ adapters
using cutadapt
Step 1: Demultiplex
Allow up to 1 mismatch in barcode
Extract UMIs
Step 3: Create reference amplicons
Use guide(s) information to create set of sequences: wt, inversion, large deletion, etc
Step 4: Alignment
Index amplicons and do global alignment using bowtie2
Step 6: Count small editing events
Get detailed small indel counts collapsed by UMI in all amplicons
Step 5: Count reads in amplicons
Get counts collapsed by UMI in all amplicons
