# Supplementary material for: UDiTaS™, a genome editing detection method for indels and genome rearrangements
Source: BMC Genomics. 2018 Mar 21;19:212. doi: 10.1186/s12864-018-4561-9 (PMC5861650; doi:10.1186/s12864-018-4561-9)
Supplement: Supplementary file 2 — Detailed Protocols for UDiTaS and AMP-Seq methods. (DOCX 237 kb) [file 12864_2018_4561_MOESM2_ESM.docx]

Supplementary Methods

**UDiTaS protocol**

**1. Equipment**

96 well PCR plates (ThermoFisher, #AB-1400-L)

Plate Seals – Empore^TM^ Sealing Tape (Sigma-Aldrich, #66881-U)

96/384-well plate spinner (ThermoFisher, 14-100-143)

Qubit 3.0 Fluorometer (ThermoFisher, #Q33216)

Veriti Thermal Cycler, 96-well (ThermoFisher, #4375786)

Agilent 2100 BioAnalyzer (Agilent, #G2939AA); Agilent High Sensitivity DNA Kit (# 5067-4626)

BluePippin (Sage Science)

1. **Bench Cleanup**

- DNAse AWAY spray (Molecular BioProducts #7002)
- Kimwipes (Kimberly Clark #34256)

1. Spray down bench and all pipettors. Wipe dry
2. **Adapter oligo annealing**

3.1 Materials:

- 96 well PCR plate
- 96 Thermal Cycler
- Top oligo (IDT): refer to appendix A for the 8-possible top oligos with the full length Illumina i5 adapter sequence
- Bottom oligo (IDT): refer to appendix A for single bottom oligo

3.2 Oligo annealing set up (Refer to Appendix A for adapter oligo info)

1. Set up in a 96 well PCR plate
2. For each 100 uL reaction, add 50 uL the top and bottom oligo

| Reagents | 100 µL reaction | Final conc. (µM) |
| --- | --- | --- |
| Top oligo (100 µM) | 50 µL | 50 |
| Bottom oligo (100 µM) | 50 µL | 50 |
| Total vol. | 100 µL |  |

1. Set the PCR thermocycler 95° C for 2 min and slow ramp down to 25° C (~12 hours)
2. Aliquot into labeled 0.7 mL tubes in single use volumes (15 or 30 µL)
3. Store at -20° C
4. **Transposome assembly**

4.1 Materials:

- DNA LoBind Tube 1.5 mL tubes (Eppendorf, #022431021)
- pTXB1-Tn5 vector was synthesized by GeneWiz. Academic institutes can purchase the vector from addgene (<https://www.addgene.org/60240/>). The vector was expressed according to Picelli, S. *et al.* *Genome Res.* **24,** 2033–2040 (2014). Final protein concentration was 1.85 mg/ml and stored at -20°C in 1 mL aliquots:
  - unassembled Tn5 storage buffer: 55%glycerol stock: 100% glycerol + 2x Tn5 dialysis buffer
  - 2x Tn5 dialysis buffer: 100 HEPES-KOH at pH7.2, 0.2M NaCl, 0.2mM EDTA, 2mM DTT, 0.2% Triton X-100, 20% glycerol
- Annealed oligos from step 3

4.2 Transposome assembly:

1. Keep Tn5 enzyme at -20°C until ready to use. In LoBind 1.5mL Eppendorf tubes, add pre-annealed oligo and transposome.

| Reagents | % reaction volume | 100 µL reaction | 200 µL reaction |
| --- | --- | --- | --- |
| Tn5 enzyme (1.85mg/mL) | 0.857 | 85.7 µL | 171.4 µL |
| Pre-annealed oligos (100µM) | 0.143 | 14.3 µL | 28.6 µL |
| Total volume (µL) | 1 | 100 µL | 200 µL |

1. Incubate at room temperature for 60 minutes
2. Test transposome activity by setting up tagmentation reactions (step 5)
3. Store aliquots in -20° C
4. **Tagmentation reaction**

5.1 Materials:

- 96 well PCR plate
- Human genomic DNA, 10 ng/µL (Promega, # G3041)
- 5X TAPS-DMF: Boston BioProducts (custom buffer)
  - 50mM TAPS-NaOH @ pH 8.5, 25 mM MgCl2 and 50% DMF (dimethylformamide)
- Transposome (8 different transposome can be generated using the bottom oligo with each of the i5 barcoded top oligos listed in Appendix A)
- DNA clean & concentrator-5 columns (Zymo Research, # D4013)

5.2 Tagmentation setup:

**NOTE:** Accurate quantification of DNA is essential for reproducible tagmentation resulting in ~1.5-2 kb products (see Appendix B for tagmentation profile)

- Samples are processed in small batches (12) to reduce any premature activity of the transposase before incubation.
- Amount of transposome may vary depending on your activity assay
- For **tagmentation activity assay**: set up reactions below doing a titration of the transposome keeping the gDNA constant (50ng). Then clean up with Zymo column and run on the Bioanalyzer to check the size of the library.

1. Preheat the thermocycler to 55° C
2. Make a mastermix with water, 5x TAPS-DMF buffer and transposome
3. Mix well but gently

| Reagents | 1 reaction |
| --- | --- |
| Nuclease free water | 4 µL |
| 5x TAPS-DMF | 4 µL |
| Genomic DNA (5 ng/µL) | 10 µL |
| Assembled transposome | 2 µL |
| Total reaction (µL) | 20 µL |

1. Aliquot 10 µL of mix to each well
2. Add 10 µL of gDNA (50 ng total) to each well
3. Pipet up and down to mix slowly. Avoid bubbles as possible
4. Seal plate and spin briefly
5. Incubate the plate in thermocycler at 55° C for 7 minutes
6. Place on ice
   1. Tagmentation reaction clean up using Zymo columns
7. Remove plate from ice and add 100 µL of DNA binding buffer (5x reaction volume)
8. Mix briefly by pipetting up and down or vortex briefly
9. Transfer mixture to Zymo-Spin column in a 2mL collection tube
10. Centrifuge for 30 sec (13,000xg); do not need to discard, flow through volume is low
11. Add 200 ul DNA Wash Buffer to the column.
12. Centrifuge for 30 sec (13,000xg); do not need to discard, flow through volume is low
13. Add another 200 µL DNA Wash Buffer to the column.
14. Centrifuge for 30 sec (15,000xg)
15. Place filter column in a clean 1.5 mL tubes
16. Add 11 µL of nuclease free water to each column
17. Incubate at room temperature for 1 minute
18. Centrifuge for 30 sec (15,000xg)
19. **Round 1 PCR**

6.1 Materials:

- 96 well PCR plate
- 2x Platinum SuperFi PCR Master mix (ThermoFisher, # 12358050)
- 5M Tetramethylammonium chloride solution (TMAC; Sigma, # T3411-500ML)
- VEGFA gene specific primer (GSP) for assay validation. This can be replaced with gene specific primer of interest:
  - VEGFA_chr6_43737470_F: IDT; 5’-GTGACTGGAGTTCAGACGTGTGCTCTTCCGATCTCAACTCCAGTCCCAAATATGTAGCTGTT-3’; Illumina i7 tail in red
- i5 amplification primer: IDT: 5’-AATGATACGGCGACCACCGAGATCTACAC- 3’
- Tagmented DNA (from step 5)

6.2 Round 1 PCR setup:

| Reagents | 1 reaction | Final concentration |
| --- | --- | --- |
| Water (SuperFi kit) | To 25 µL |  |
| 2x SuperFi master mix | 12.5 µL | 1x |
| TMAC (0.5M) | 1.5 µL | 30 mM |
| GSP (10 µM) | 1.25 µL | 500 nM |
| i5 primer | 0.625 µL | 250 nM |
| Tagmented DNA | 9 µL |  |
| Total reaction | 25 µL |  |

1. Make a master mix for each gene specific primer (GSP), add components as listed aboveAdd 15.875 µL of the mix into the wells of the 96 well plate
2. Add 9.125 µL of the Zymo cleaned tagmentation products to each well
3. Mix well and slowly to avoid bubbles
4. Seal plate and spin briefly
5. Place the plate in thermocycler with the following program:

| Step | Temp (^o^C) | Time | Cycle # |
| --- | --- | --- | --- |
| Initial Denaturation | 98 | 2 min | 1 |
| Denature | 98 | 10 sec | 12 |
| Anneal | 65 | 10 sec |  |
| Extend | 72 | 90 sec |  |
| Final Extension | 72 | 5 min | 1 |
| Hold | 4 |  | 1 |

6.3 Clean up using Agencourt Ampure XP SPRI beads (0.9X reaction volume)

6.3.1 Materials:

- Agencourt AMPure XP (#A63882)
- DynaMag™-96 Side Magnet (#12331D)
- 200 Proof Pure Ethanol (KOPTEC #V1016)
- DNase-RNase Free Water Non-DEPC Treated (Boston Bioproducts #R-100DR)

6.3.2 Ampure XP SPRI beads clean up

1. Add 22.5 µL of Ampure XP beads to each well (25 µL x 0.9x reaction volume = 22.5 µL)
2. Mix beads with reaction by pipetting up and down 15 times
3. Let stand in room temperature for 5 minutes
4. Place the plate on the magnet and let stand for 5 minutes or until the solution is clear
5. Remove and discard supernatant
6. Wash two times with 200 µL of 70% ethanol (made fresh before use). Let the ethanol sit in the beads for 30 seconds then remove ethanol.
7. Let the beads air dry for 3-5 minutes (until the pellet is not glossy).
8. Elute with 11 µL of water (use nuclease free water)
9. **Round 2 PCR -** refer to Appendix A for barcoded PCR 2 primers

7.1 Materials:

- 2x Platinum SuperFi PCR Master mix (Thermo Fisher #12358050)
- i7 barcoded primer (see Appendix A)

• i5 amplification primer: IDT: 5’-AATGATACGGCGACCACCGAGATCTACAC- 3’

- Round 1 PCR product
  1. Round 2 PCR set up

1. In a new 1.5mL tube, make a master mix with water, P5 primer and 2X Platinum SuperFi PCR master mix

| Reagents | 1 reaction | Final concentration |
| --- | --- | --- |
| Water (SuperFi kit) | 5 µL |  |
| 2x SuperFi master mix | 25 µL | 1x |
| i5 amplification primer (10 µM) | 2.5 µL | 500 nM |
| 1^st^ round PCR product | 10 µL |  |
| i7 barcode primer (100 µM) | 7.5 µL | 15 µM |
| Total reaction | 50 µL |  |

1. Aliquot 32.5 µL into each well with round 1 PCR product
2. Add 7.5 µL of unique i7 barcode primer to each well
3. Add 10 µL of purified Round 1 PCR product to each well
4. Mix well
5. Place plate in thermocycler and run the following program:

| Step | Temp (^o^C) | Time | Cycle # |
| --- | --- | --- | --- |
| Initial Denaturation | 98 | 2 min | 1 |
| Denature | 98 | 10 sec | 15 |
| Anneal | 65 | 10 sec |  |
| Extend | 72 | 90 sec |  |
| Final Extension | 72 | 5 min | 1 |
| Hold | 4 |  | 1 |

7.3 Clean up using Agencourt Ampure XP SPRI bead (0.9X reaction volume)

7.3.1 Materials:

- Agencourt AMPure XP (#A63882)
- DynaMag™-96 Side Magnet (#12331D)
- 200 Proof Pure Ethanol (KOPTEC #V1016)
- DNase-RNase Free Water Non-DEPC Treated (Boston Bioproducts #R-100DR)
  - 1. Round 2 PCR Ampure Cleanup:

1. Add 45 µL of Ampure XP beads to each well (50 µL x 0.9x = 45 µL)
2. Mix beads with reaction by pipetting up and down 15 times.
3. Let stand in room temperature for 5 minutes.
4. Place the plate on the magnet and let stand for 5 minutes or until the solution is clear.
5. Remove and discard supernatant.
6. Wash two times with 200 µL of 70% ethanol (made fresh before use). Let the ethanol sit in the beads for 30 seconds then remove ethanol.
7. Let the beads air dry for 3-5 minutes (until the pellet is not glossy).
8. Elute with 25 µL of 1x low TE (10 mM Tris, 0.1 mM EDTA)
9. **QC with Bioanalyzer and Qubit**

8.1 Bioanalyzer QC following manufacturer’s specifications

<http://www.agilent.com/cs/library/usermanuals/Public/G2938-90322_HighSensitivityDNA_QSG.pdf>

8.2 Qubit Quantification following manufacturer’s specifications for the dsDNA High Sensitivity kit

<https://tools.thermofisher.com/content/sfs/manuals/Qubit_dsDNA_HS_Assay_UG.pdf>

1. **Pool and concentrate samples**

9.1 Materials

- Agencourt AMPure XP (#A63882)
- DynaMag™-96 Side Magnet (#12331D)
- 200 Proof Pure Ethanol (KOPTEC #V1016)
- DNase-RNase Free Water Non-DEPC Treated (Boston Bioproducts #R-100DR)

9.2 Pooling and SPRI clean up

1. Calculate the molar amount of each product based on the concentration from qubit and the size from the bioanalyzer.
2. Pool equimolar amounts of each product into a 1 mL Eppendorf tube.
3. Clean and concentrate pool using Agencourt Ampure XP SPRI beads (0.9x)
   1. Add 0.9 times the reaction volume of Ampure XP beads
   2. Mix beads with reaction by pipetting up and down 15 times
   3. Let stand in room temperature for 5 minutes
   4. Place the plate on the magnet and let stand for 5 minutes or until the solution is clear
   5. Remove and discard supernatant
   6. Wash two times with 200 µL of 70% ethanol (made fresh before use). Let the ethanol sit in the beads for 30 seconds then remove ethanol
   7. Let the beads air dry for 3-5 minutes (until the pellet is not glossy)
   8. Elute with 35 µL of 1x low TE
   9. Set 2 µL aside for QC. Proceed with size selection with remaining
4. **Size selection using Sage BluePippin following manufacturer’s specifications**

10.1 Reagents

- - - BluePippin Gel cassettes: 1.5% agarose, 250bp – 1.5kb (Sage Science)

10.2 Size Selection (400-850bp)

- Create size selection protocol for 400-850bp on all lanes
- Load only 1.5 µg of product per lane (run multiples lanes if necessary)

**11. Clean up size selected library using Ampure XP SPRI beads (0.9x reaction volume)**

11.1 Materials:

- Agencourt AMPure XP (#A63882)
- DynaMag™-96 Side Magnet (#12331D)
- 200 Proof Pure Ethanol (KOPTEC #V1016)
- DNase-RNase Free Water Non-DEPC Treated (Boston Bioproducts #R-100DR)

11.2 Ampure XP setup

1. Add 0.9 times reaction volume of size selected product from BluePippin
2. Mix beads with reaction by pipetting up and down 15 times
3. Let stand in room temperature for 5 minutes
4. Place the plate on the magnet and let stand for 5 minutes or until the solution is clear
5. Remove and discard supernatant
6. Wash two times with 200 µL of 70% ethanol (made fresh before use). Let the ethanol sit in the beads for 30 seconds then remove ethanol
7. Let the beads air dry for 3-5 minutes (until the pellet is not glossy)
8. Elute with 25 ul of 1x low TE
9. **Quantification of final size selected library**
   1. KAPA library quant qPCR kit
      - KAPA library quant kit (illumina) Universal qPCR mix; cat# KK4824
      - Vortex to mix all reagents well
      - Dilute sample according to the table below:

| Library dilution | dilution factor |
| --- | --- |
| 1 | 1:10 |
| 2 | 1:100 |
| 3 | 1:1,000 |
| 4 | 1:10,000 |
| 5 | 1:100,000 |
| 6 | 1:1,000,000 |
| 7 | 1:2,000,000 |

1. Set up KAPA library quant qPCR according to the manual
2. Add 6 ul of KAPA master mix into each well
3. Use 4 ul of each dilution or standard in triplicate
4. Place on the qPCR instrument (2 hours)

| Temp (^o^C) | Time | Cycle # |
| --- | --- | --- |
| 95 | 5 min | 1 |
| 95 | 30 sec | 35 |
| 60 | 45 |  |
| Melt curve | | |

1. Use the KAPA analysis excel sheet provided online to copy the Cq values and the size of the library from the bioanalyzer to calculate the nM concentration of the sample
   1. QC final pooled library before and after size selection on Agilent Bioanalyzer
2. Dilute all samples 1:10 before running on the Bioanalzyer high sensitivity chip
3. Obtain the median size of the library

   Example:
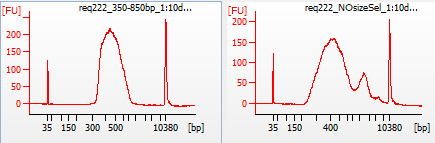


**13. MiSeq Sequencing**

13.1 Follow manufacturer’s specifications for loading library on MiSeq

1. Load 10 pM denatured library with 15% PhiX
2. Generate MiSeq run sheet:
   1. Read 1: 300 bases
   2. Index read 1: 8 bases
   3. Read 2: 300 bases
   4. Index read 2: 18 bases

**Appendix A:**

Adapter oligo sequences used to anneal (step 3) and complexing with the Tn5: Oligos were ordered from Integrated DNA Technologies (IDT)

| **Oligo Name** | **sequence 5'-3'** |
| --- | --- |
| Tn5-A bottom | [Phos]CTGTCTCTTATACA[ddC] |
| i5_N501_UMI_Tn5-A | AATGATACGGCGACCACCGAGATCTACACTAGATCGCNNNNNNNNNNTCGTCGGCAGCGTCAGATGTGTATAAGAGACAG |
| i5_N502_UMI_Tn5-A | AATGATACGGCGACCACCGAGATCTACACCTCTCTATNNNNNNNNNNTCGTCGGCAGCGTCAGATGTGTATAAGAGACAG |
| i5_N503_UMI_Tn5-A | AATGATACGGCGACCACCGAGATCTACACTATCCTCTNNNNNNNNNNTCGTCGGCAGCGTCAGATGTGTATAAGAGACAG |
| i5_N504_UMI_Tn5-A | AATGATACGGCGACCACCGAGATCTACACAGAGTAGANNNNNNNNNNTCGTCGGCAGCGTCAGATGTGTATAAGAGACAG |
| i5_N505_UMI_Tn5-A | AATGATACGGCGACCACCGAGATCTACACGTAAGGAGNNNNNNNNNNTCGTCGGCAGCGTCAGATGTGTATAAGAGACAG |
| i5_N506_UMI_Tn5-A | AATGATACGGCGACCACCGAGATCTACACACTGCATANNNNNNNNNNTCGTCGGCAGCGTCAGATGTGTATAAGAGACAG |
| i5_N507_UMI_Tn5-A | AATGATACGGCGACCACCGAGATCTACACAAGGAGTANNNNNNNNNNTCGTCGGCAGCGTCAGATGTGTATAAGAGACAG |
| i5_N508_UMI_Tn5-A | AATGATACGGCGACCACCGAGATCTACACCTAAGCCTNNNNNNNNNNTCGTCGGCAGCGTCAGATGTGTATAAGAGACAG |

| **Oligo Name** | **i5 Barcode Sequence** | **i5 Barcode READ (Tx10 for UMI)** |
| --- | --- | --- |
| i5_N501_UMI_Tn5-A | TAGATCGC | TAGATCGCTTTTTTTTTT |
| i5_N502_UMI_Tn5-A | CTCTCTAT | CTCTCTATTTTTTTTTTT |
| i5_N503_UMI_Tn5-A | TATCCTCT | TATCCTCTTTTTTTTTTT |
| i5_N504_UMI_Tn5-A | AGAGTAGA | AGAGTAGATTTTTTTTTT |
| i5_N505_UMI_Tn5-A | GTAAGGAG | GTAAGGAGTTTTTTTTTT |
| i5_N506_UMI_Tn5-A | ACTGCATA | ACTGCATATTTTTTTTTT |
| i5_N507_UMI_Tn5-A | AAGGAGTA | AAGGAGTATTTTTTTTTT |
| i5_N508_UMI_Tn5-A | CTAAGCCT | CTAAGCCTTTTTTTTTTT |

P5 / i5 oligo sequence for Round 1 and 2 PCR:

| **Oligo Name** | **sequence 5'-3'** |
| --- | --- |
| i5 | AATGATACGGCGACCACCGAGATCTACAC |

i7 Barcoded oligo sequences for Round 2 PCR:

| **Oligo Name** | **Sequence (5'-3')** | **i7 BC Seq** | **i7 BC READ** |
| --- | --- | --- | --- |
| i7_N701_SBS12 | CAAGCAGAAGACGGCATACGAGATAGCGGAATGTGACTGGAGTTCAGACGTGT | AGCGGAAT | ATTCCGCT |
| i7_N702_SBS12 | CAAGCAGAAGACGGCATACGAGATGATCATGCGTGACTGGAGTTCAGACGTGT | GATCATGC | GCATGATG |
| i7_N703_SBS12 | CAAGCAGAAGACGGCATACGAGATAAGACGGAGTGACTGGAGTTCAGACGTGT | AAGACGGA | TCCGTCTT |
| i7_N704_SBS12 | CAAGCAGAAGACGGCATACGAGATCGAGTCCTGTGACTGGAGTTCAGACGTGT | CGAGTCCT | AGGACTCG |
| i7_N705_SBS12 | CAAGCAGAAGACGGCATACGAGATTCCTCAGGGTGACTGGAGTTCAGACGTGT | TCCTCAGG | CCTGAGGA |
| i7_N706_SBS12 | CAAGCAGAAGACGGCATACGAGATGTACGGATGTGACTGGAGTTCAGACGTGT | GTACGGAT | ATCCGTAC |
| i7_N707_SBS12 | CAAGCAGAAGACGGCATACGAGATCATCTCTCGTGACTGGAGTTCAGACGTGT | CATCTCTC | GAGAGATG |
| i7_N710_SBS12 | CAAGCAGAAGACGGCATACGAGATGTCGGAGCGTGACTGGAGTTCAGACGTGT | GTCGGAGC | GCTCCGAC |
| i7_N711_SBS12 | CAAGCAGAAGACGGCATACGAGATACGGAGAAGTGACTGGAGTTCAGACGTGT | ACGGAGAA | TTCTCCGT |
| i7_N712_SBS12 | CAAGCAGAAGACGGCATACGAGATAGGAGATGGTGACTGGAGTTCAGACGTGT | AGGAGATG | CATCTCCT |
| i7_N714_SBS12 | CAAGCAGAAGACGGCATACGAGATAGTACTCGGTGACTGGAGTTCAGACGTGT | AGTACTCG | CGAGTACT |
| i7_N715_SBS12 | CAAGCAGAAGACGGCATACGAGATGGACTCTAGTGACTGGAGTTCAGACGTGT | GGACTCTA | TAGAGTCC |

**Appendix B: Tagmentation Profile viewed on the Agilent Bioanalyzer**

50 ng Promega gDNA using 2 µL of transposome in 20 µL tagmentation reaction and incubated in a preheated thermal cycler at 55^o^C for 7 min


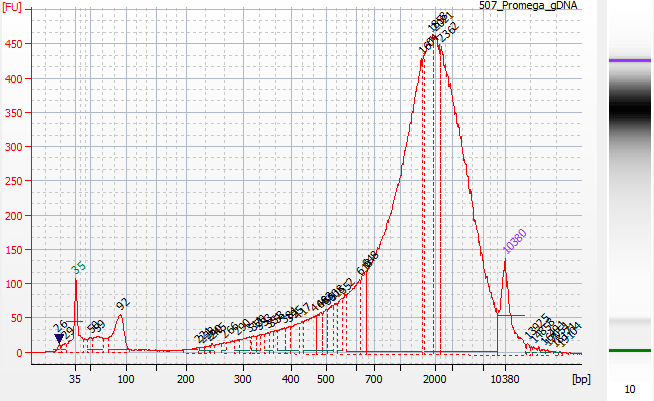


**AMP-seq protocol**

A modified AMP-seq protocol previously described (Tsai, S. Q. *et al.* GUIDE-seq enables genome-wide profiling of off-target cleavage by CRISPR-Cas nucleases. *Nat. Biotechnol.* **33,** 187–197 (2014)) was used to generate the AMP-seq libraries.

1. **Equipment**

96 well PCR plates (ThermoFisher, #AB-1400-L)

Plate Seals – Empore^TM^ Sealing Tape (Sigma-Aldrich, #66881-U)

96/384-well plate spinner (ThermoFisher, 14-100-143)

Qubit 3.0 Fluorometer (ThermoFisher, #Q33216)

Veriti Thermal Cycler, 96-well (ThermoFisher, #4375786)

Agilent 2100 BioAnalyzer (Agilent, #G2939AA); Agilent High Sensitivity DNA Kit (# 5067-4626)

BluePippin (Sage Science)

Covaris M220 instrument

1. **Materials:**

- End repair Kit (Enzymatics, Y9140-LC-L)
- Ligation Kit: (Enzymatics, L6030-LC-L)
- Platinum Taq: (Invitrogen, 10966-026)
- Nuclease free water
- 1x TE (10mM Tris, 1mM EDTA, pH 8.0)
- Pre-annealed Y adapters
- TetraMethylAmmonium Chloride (TMAC) 5M
- Agencourt AMPure XP (#A63882)
- DynaMag™-96 Side Magnet (#12331D)
- 200 Proof Pure Ethanol (KOPTEC #V1016)
- DNase-RNase Free Water Non-DEPC Treated (Boston Bioproducts #R-100DR)
- **AMP-seq adapter sequences – Oligos ordered from IDT**

| **Y-Adapters** | **Sequence 5’🡪 3’** |
| --- | --- |
| OLI2909 | AATGATACGGCGACCACCGAGATCTACACACTGCATANNWNNWNNACACTCTTTCCCTACACGACGCTCTTCCGATC*T |
| OLI2910 | ATGATACGGCGACCACCGAGATCTACACAAGGAGTANNWNNWNNACACTCTTTCCCTACACGACGCTCTTCCGATC*T |
| bottom | [Phos]GATCGGAAGAGC*C*A |

* Phosphorothioate bond

100uM of each top and bottom oligos are heated to 95^o^C for 1sec, 60^o^C for 1sec and ramped down 2^o^C/min to 4^o^C and hold at 4^o^C.

1. **Method:**

50 ng of into mouse genomic DNA containing the CEP290 plasmids (PLA370, PLA367, PLA371, PLA368, PLA372, PLA369) ranging from ~714,000 to 2,200 genome equivalents was used to generate AMP-seq libraries. The DNA (50ng in 130uL total TE buffer) was sheared using the Covaris M220 (shearing conditions- peak power:50, cycles/burst:200, duration:60s, duty factor:10) to obtain 500bp fragments. The sample was concentrated using AmPure XP beads (130uL) following the manufacturer’s specifications and eluted in 15uL 1x TE.

The DNA fragment were end repaired as follows:

| **Reagent** | **Volume/reaction (µL)** |
| --- | --- |
| Nuclease free water | 0.5 |
| dNTP (5mM) | 1 |
| SLOW Ligation buffer (10x) | 2.5 |
| End repair mix low concentration | 2 |
| 10x Buffer for Platinum Taq Mg2+ free | 2 |
| Platinum Taq 5U/uL) | 0.5 |
| Fragmented DNA | 14 |
| Total | 22.5 |

End Repair thermocycler program used was: 12°C for 15min, 37°C for 15min; 72°C for 15min; hold at 4°C. Pre-annealed unique Illumina adapters (1uL at 10uM adapters: either OLI2909, OLI2910) were added to each reaction along with 2uL of T4 DNA ligase and incubated on thermal cycler (16°C for 30min, 22°C for 30min, hold at 4°C). The reaction was cleaned using AmPure XP beads (0.9X volume, 22.5uL) and eluted in 12uL 1x TE buffer.

1. **Round 1 PCR:**

The CEP290 library was amplified with gene specific primer (OLI6062):

| **Reagent** | **Volume/reaction (µL)** |
| --- | --- |
| Nuclease free water | 11.15 |
| 10x Buffer for Platinum Taq Mg2+ free | 3 |
| 50mM Mg2+ | 1.2 |
| Platinum Taq (5U/uL) | 0.3 |
| dNTP 10mM | 0.6 |
| TMAC 0.5M | 1.5 |
| i5 amplification primer (10uM) | 0.75 |
| Gene specific primer (10uM) | 1.5 |
| Adapter ligated DNA | 10 |
| Total | 30 |

Cycling conditions:

| **Temp** | **Time** | **Cycle** |
| --- | --- | --- |
| 95 degree C | 5 minutes |  |
| 95 degree C | 30 seconds | 15x |
| 70 degree C, -1 C per cycle | 2 minutes | 15x |
| 72 degree C | 30 seconds | 15x |
| 95 degree C | 30 seconds | 10x |
| 55 degree C | 1 minute | 10x |
| 72 degree C | 30 seconds | 10x |
| 4 degree C | hold |  |

PCR products were cleaned using AmPure XP beads (1.2x volume, 36uL) following the manufacturer’s specifications and eluted in 17uL 1x TE.

1. **Round 2 PCR:**

The CEP290 library was amplified with i7 barcode primers (i7_N701_SBS12, i7_N702_SBS12):

| **Reagent** | **Volume/reaction (µL)** |
| --- | --- |
| Nuclease free water | 4.65 |
| 10x Buffer for Platinum Taq Mg2+ free | 3 |
| 50mM Mg2+ | 1.2 |
| Platinum Taq (5U/uL) | 0.3 |
| dNTP 10mM | 0.6 |
| TMAC 0.5M | 1.5 |
| i5 amplification primer (10uM) | 0.75 |
| i7_barcode_SBS12 (10uM) | 3 |
| Round 1 PCR product | 15 |
| Total | 30 |

Cycling conditions:

| **Temp** | **Time** | **Cycle** |
| --- | --- | --- |
| 95 degree C | 5 minutes |  |
| 95 degree C | 30 seconds | 15x |
| 70 degree C, -1 C per cycle | 2 minutes | 15x |
| 72 degree C | 30 seconds | 15x |
| 95 degree C | 30 seconds | 10x |
| 55 degree C | 1 minute | 10x |
| 72 degree C | 30 seconds | 10x |
| 4 degree C | hold |  |

PCR products were cleaned using AmPure XP beads (0.7x volume, 21uL) following the manufacturer’s specifications and eluted in 25uL 1x TE.

1. **QC with Bioanalyzer and Qubit**

8.1 Bioanalyzer QC following manufacturer’s specifications

http://www.agilent.com/cs/library/usermanuals/Public/G2938-90322_HighSensitivityDNA_QSG.pdf8.2 Qubit Quantification following manufacturer’s specifications for the dsDNA High Sensitivity kit

<https://tools.thermofisher.com/content/sfs/manuals/Qubit_dsDNA_HS_Assay_UG.pdf>

1. **Pool and concentrate samples**

9.1 Materials

- Agencourt AMPure XP (#A63882)
- DynaMag™-96 Side Magnet (#12331D)
- 200 Proof Pure Ethanol (KOPTEC #V1016)
- DNase-RNase Free Water Non-DEPC Treated (Boston Bioproducts #R-100DR)

9.2 Pooling and SPRI clean up

1. Calculate the molar amount of each product based on the concentration from qubit and the size from the bioanalyzer.
2. Pool equimolar amounts of each product into a 1 mL Eppendorf tube.
3. Clean and concentrate pool using Agencourt Ampure XP SPRI beads (0.9x)
   1. Add 0.9 times the reaction volume of Ampure XP beads
   2. Mix beads with reaction by pipetting up and down 15 times
   3. Let stand in room temperature for 5 minutes
   4. Place the plate on the magnet and let stand for 5 minutes or until the solution is clear
   5. Remove and discard supernatant
   6. Wash two times with 200 µL of 70% ethanol (made fresh before use). Let the ethanol sit in the beads for 30 seconds then remove ethanol
   7. Let the beads air dry for 3-5 minutes (until the pellet is not glossy)
   8. Elute with 25 µL of 1x low TE
   9. Set 2 µL aside for QC. Proceed with size selection with remaining

**Size selection using Sage BluePippin following manufacturer’s specifications**

10.1 Reagents

- - - BluePippin Gel cassettes: 1.5% agarose, 250bp – 1.5kb (Sage Science)

10.2 Creating a Size selection protocol for the BluePippin 1.5% internal standard R2 (400-850bp)

<http://www.sagescience.com/wp-content/uploads/2011/10/Quick-Guide-BDF1510-marker-R22.pdf>

- Create size selection protocol for 400-850bp on all lanes
- Load only 1.5 µg of product per lane (run multiples lanes if necessary)

**11. Clean up size selected library using Ampure XP SPRI beads (0.9x reaction volume)**

11.1 Materials:

- Agencourt AMPure XP (#A63882)
- DynaMag™-96 Side Magnet (#12331D)
- 200 Proof Pure Ethanol (KOPTEC #V1016)
- DNase-RNase Free Water Non-DEPC Treated (Boston Bioproducts #R-100DR)

11.2 Ampure XP setup

1. Add 0.9 times reaction volume of size selected product from BluePippin
2. Mix beads with reaction by pipetting up and down 15 times
3. Let stand in room temperature for 5 minutes
4. Place the plate on the magnet and let stand for 5 minutes or until the solution is clear
5. Remove and discard supernatant
6. Wash two times with 200 µL of 70% ethanol (made fresh before use). Let the ethanol sit in the beads for 30 seconds then remove ethanol
7. Let the beads air dry for 3-5 minutes (until the pellet is not glossy)
8. Elute with 25 ul of 1x low TE
9. **Quantification of final size selected library**
   1. KAPA library quant qPCR kit
      - KAPA library quant kit (Illumina) Universal qPCR mix; cat# KK4824
      - Vortex to mix all reagents well
      - Dilute sample according to the table below:

| Library dilution | dilution factor | Sample (µL) | Qiagne EB buffer (µL) | Total (µL) |
| --- | --- | --- | --- | --- |
| 1 | 1:10 | 10 | 90 | 100 |
| 2 | 1:100 | 10 | 90 | 100 |
| 3 | 1:1,000 | 10 | 90 | 100 |
| 4 | 1:10,000 | 10 | 90 | 100 |
| 5 | 1:100,000 | 10 | 90 | 100 |
| 6 | 1:1,000,000 | 10 | 90 | 100 |
| 7 | 1:2,000,000 | 50 | 50 | 100 |

1. Set up KAPA library quant qPCR according to the manual
2. Add 6 ul of KAPA master mix into each well
3. Use 4 ul of each dilution or standard in triplicate
4. Place on the qPCR instrument (2 hours)
5. Use the KAPA analysis excel sheet provided online to copy the Cq values and the size of the library from the bioanalyzer to calculate the nM concentration of the sample
   1. QC final pooled library before and after size selection on Agilent Bioanalyzer
6. Dilute all samples 1:10 before running on the Bioanalzyer high sensitivity chip
7. Obtain the median size of the library

Example:


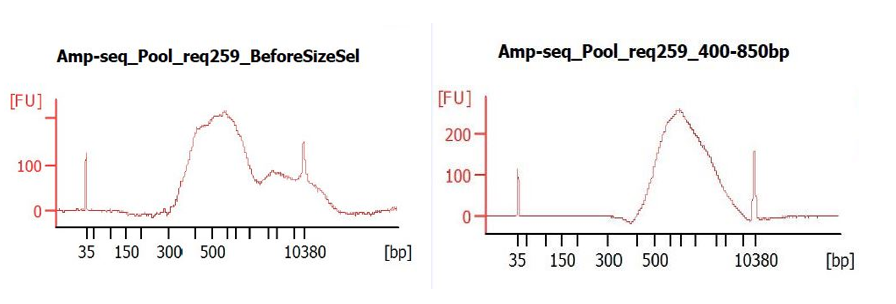


**13. MiSeq Sequencing**

13.1 Follow manufacturer’s specifications for loading library on MiSeq using a Illumina MiSeq Reagent Kit V3

1. Load 10 pM denatured library with 10-15% PhiX
2. Generate MiSeq run sheet:
   1. Read 1: 300 bases
   2. Index read 1: 8 bases
   3. Read 2: 300 bases
   4. Index read 2: 16 bases
