# Supplementary material for: UDiTaS™, a genome editing detection method for indels and genome rearrangements
Source: BMC Genomics. 2018 Mar 21;19:212. doi: 10.1186/s12864-018-4561-9 (PMC5861650; doi:10.1186/s12864-018-4561-9)
Supplement: Supplementary file 3 — Figure S2. Example editing events. Examples of various editing events in the U-2 OS bulk editing experiment shown in the Integrated Genome Viewer (IGV) [26, 27]. A schematic on top of each view depicts the observed editing event. Reads colored in red/blue were aligned to the top/bottom genomic reference DNA sequence. Note that small indels are observed in addition to the junctions formed from the larger structural changes. These indels likely arose due to repair pathway activity prior to rearrangement. a. 323 site small indels. b. 323-64 large desired 1.1 kb deletion junction. c. 323-64 large desired 1.1 kb inversion junction. d. 323 homologous junction. (PPTX 147 kb) [file 12864_2018_4561_MOESM3_ESM.pptx]

## Slide 1
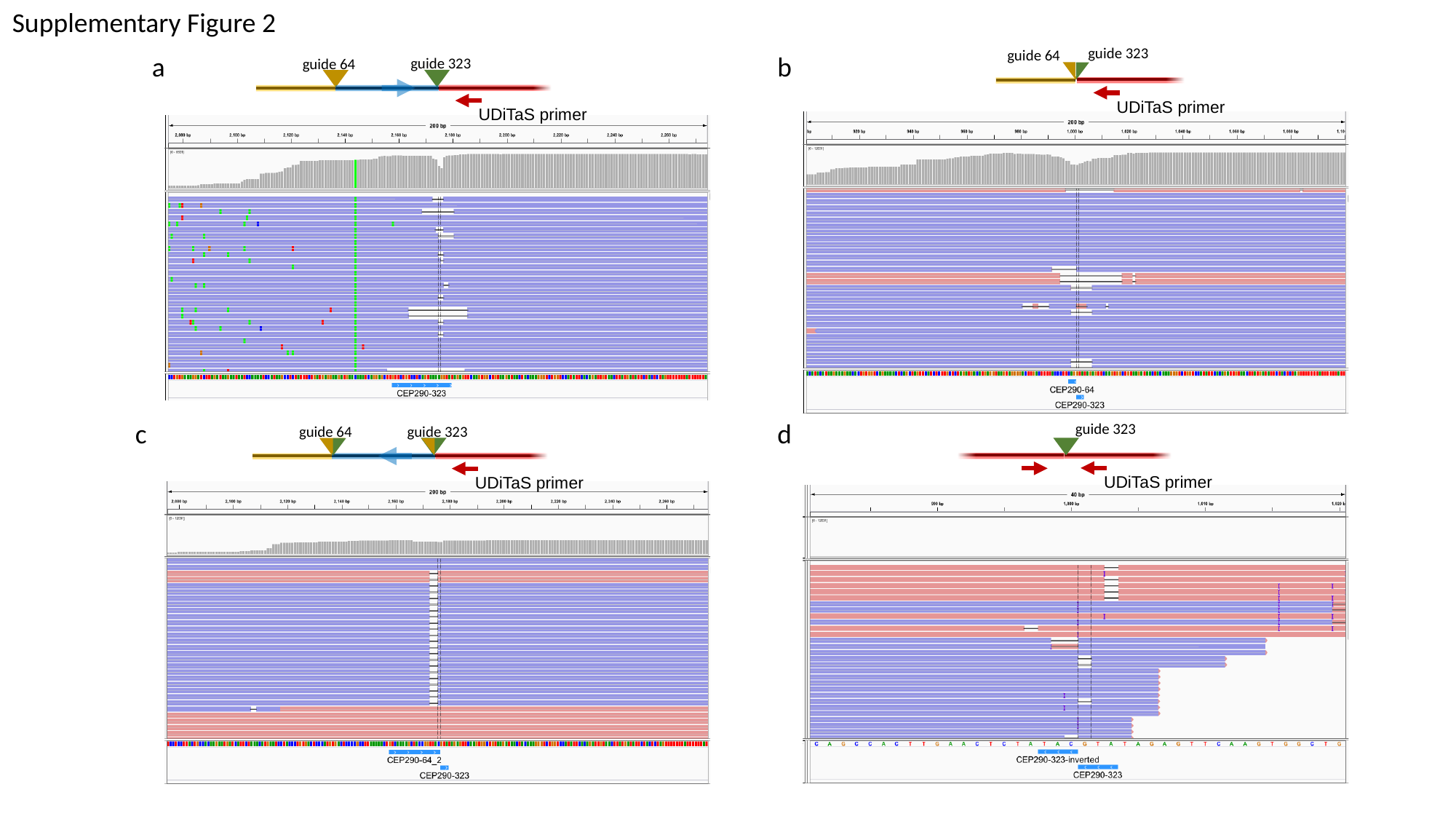

Supplementary Figure 2
guide 323
guide 64
UDiTaS primer
a
b
guide 323
guide 64
UDiTaS primer
c
d
guide 323
UDiTaS primer
guide 323
guide 64
UDiTaS primer
