# Supplementary figures and images for: UDiTaS™, a genome editing detection method for indels and genome rearrangements
Source: BMC Genomics. 2018 Mar 21;19:212. doi: 10.1186/s12864-018-4561-9 (PMC5861650; doi:10.1186/s12864-018-4561-9)

## Slide 1
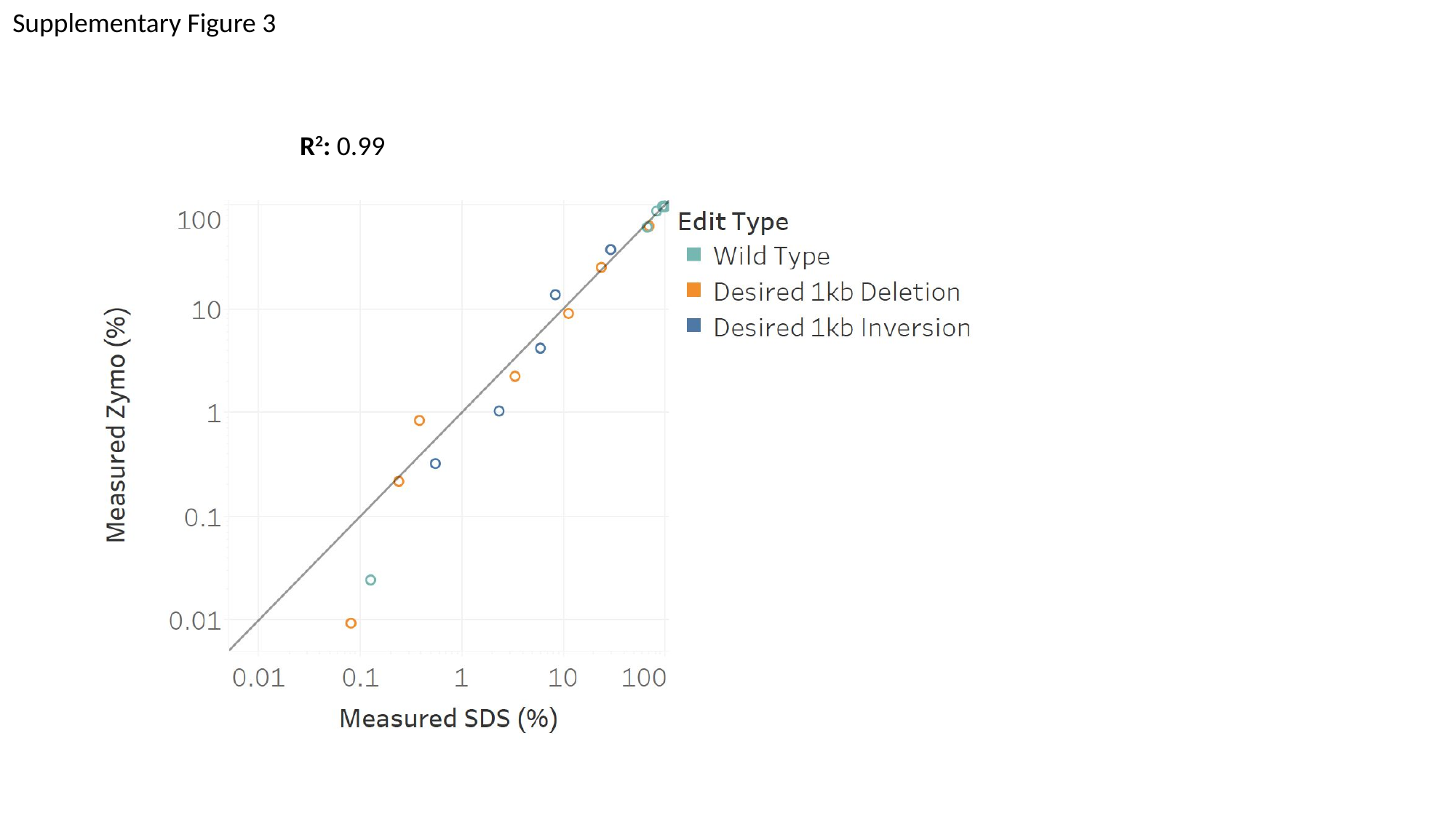

Supplementary Figure 3
R2: 0.99

Supplement: Supplementary file 4 — Figure S3. UDiTaS reproducibility. Identical samples were run in UDiTaS using either SDS addition or Zymo column purification after tagmentation. Measured values for the various constructs are reproducible and highly correlated across a wide range of concentrations. (PPTX 3928 kb) [file 12864_2018_4561_MOESM4_ESM.pptx]

## Slide 1
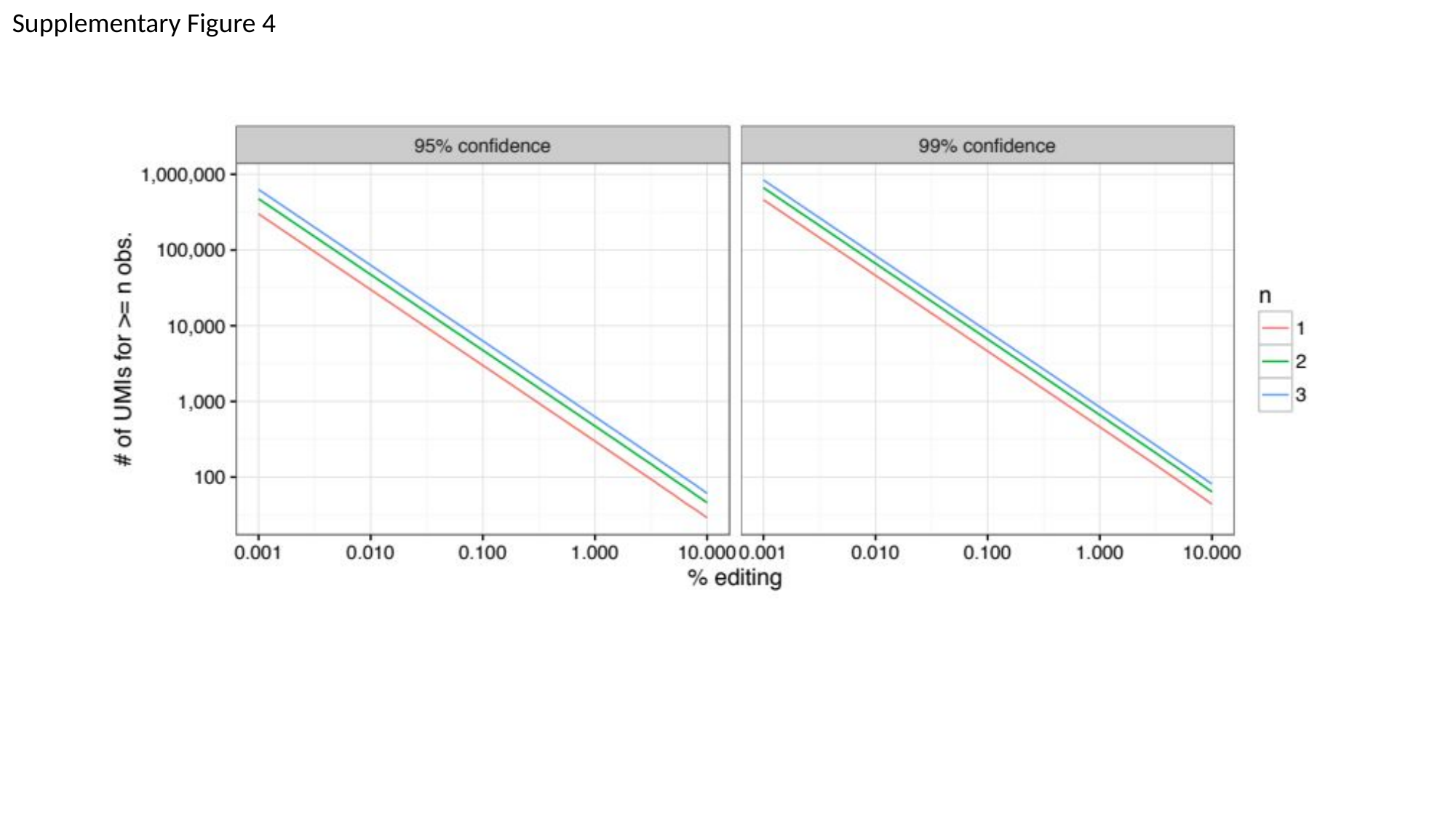

Supplementary Figure 4

Supplement: Supplementary file 5 — Figure S4. Binomial power calculation applied to UDiTaS. A simulated binomial distribution, plotting editing frequency (e.g.: probability of success) vs. number of unique molecular identifiers (e.g.: trials) for a given number of expected observations (1, 2, or 3). Graphs on the left are 95% confidence and right 99% confidence. (PPTX 86 kb) [file 12864_2018_4561_MOESM5_ESM.pptx]

## Slide 1
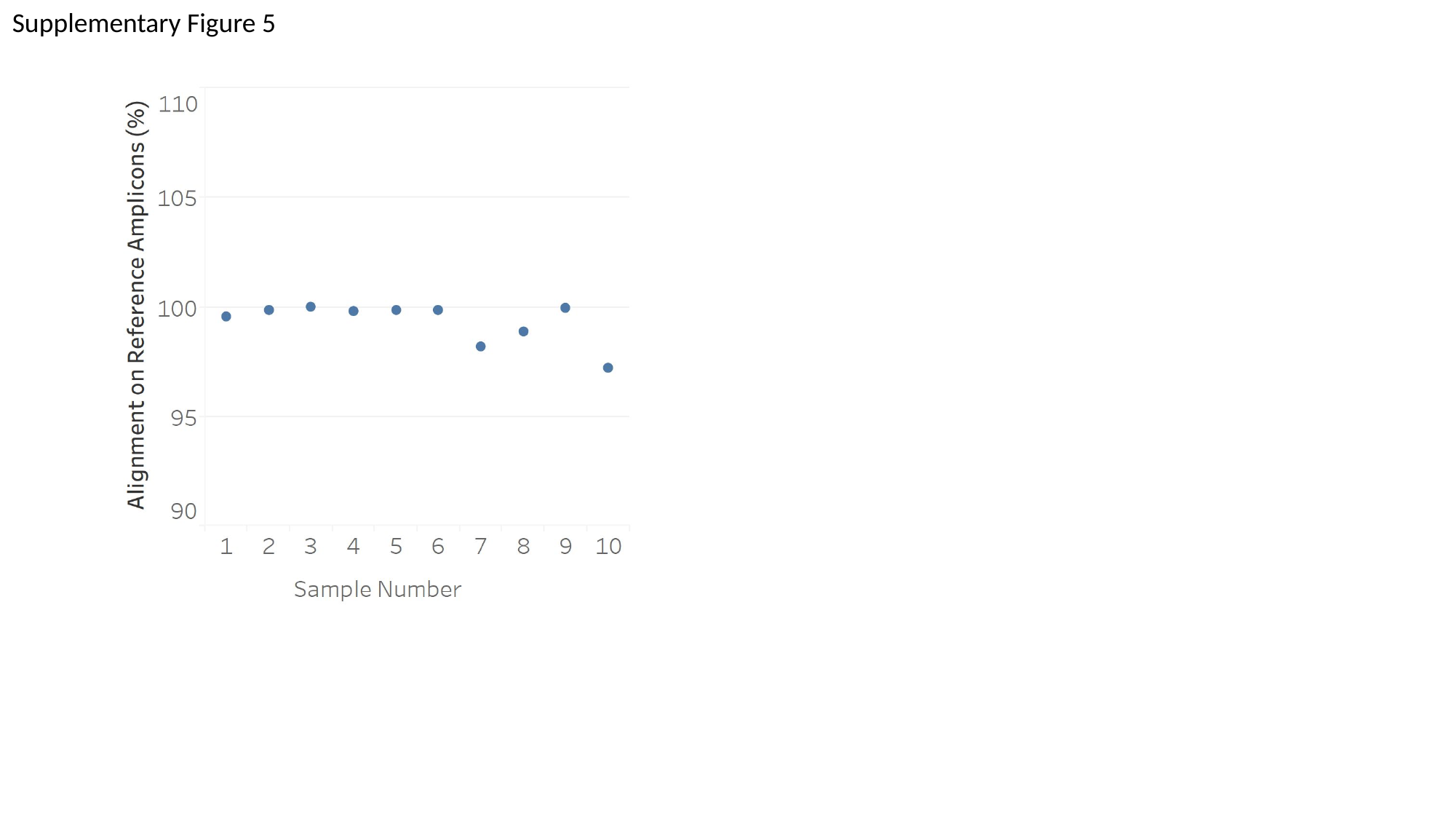

Supplementary Figure 5

Supplement: Supplementary file 6 — Figure S5. Genome mapping rates for UDiTaS. Individual reads map to the expected genome site with high frequency indicating the robustness of the assay. Ten distinct samples for primer OLI6062 are plotted on the x-axis and the y-axis shows the percentage or reads mapping to the expected reference amplicon for each sample. (PPTX 3767 kb) [file 12864_2018_4561_MOESM6_ESM.pptx]

## Slide 1
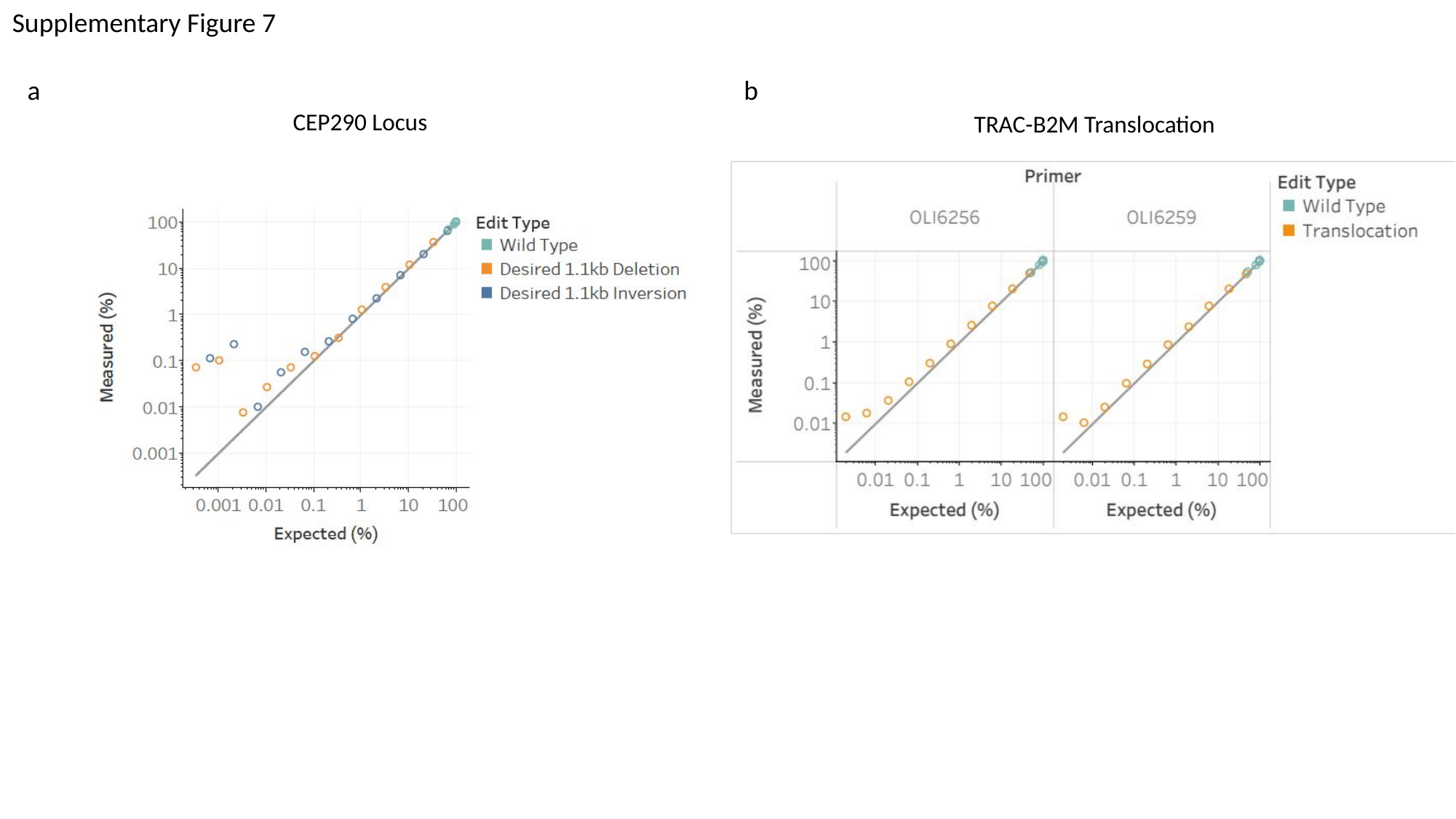

Supplementary Figure 7
a
b
CEP290 Locus
TRAC-B2M Translocation

Supplement: Supplementary file 8 — Figure S7. UDiTaS characterization of plasmid standards without carrier DNA. To ensure that the carrier mouse genomic DNA was not influencing the UDiTaS reaction, additional sets of UDiTaS reactions were run with plasmids in the absence of any carrier DNA. a. CEP290 plasmids with the Wild Type, Large Deletion, and Large Insertion (PLA379, PLA367, and PLA370) and b. B2M-TRAC plasmids with the B2M, TRAC, and both balanced translocations (PLA377, PLA378, PLA365, and PLA366) were diluted as described in the methods. The DNA plasmids mixtures were process through UDiTaS and the analysis pipeline. Plotted is the expected frequency for a given structural variant vs. measured frequency for a structural variant (x = y is the grey line). Accuracy and linearity appear to be excellent for both loci with all four primers, with an LLOD of ~ 0.01%-0.1%. (PPTX 991 kb) [file 12864_2018_4561_MOESM8_ESM.pptx]

## Slide 1
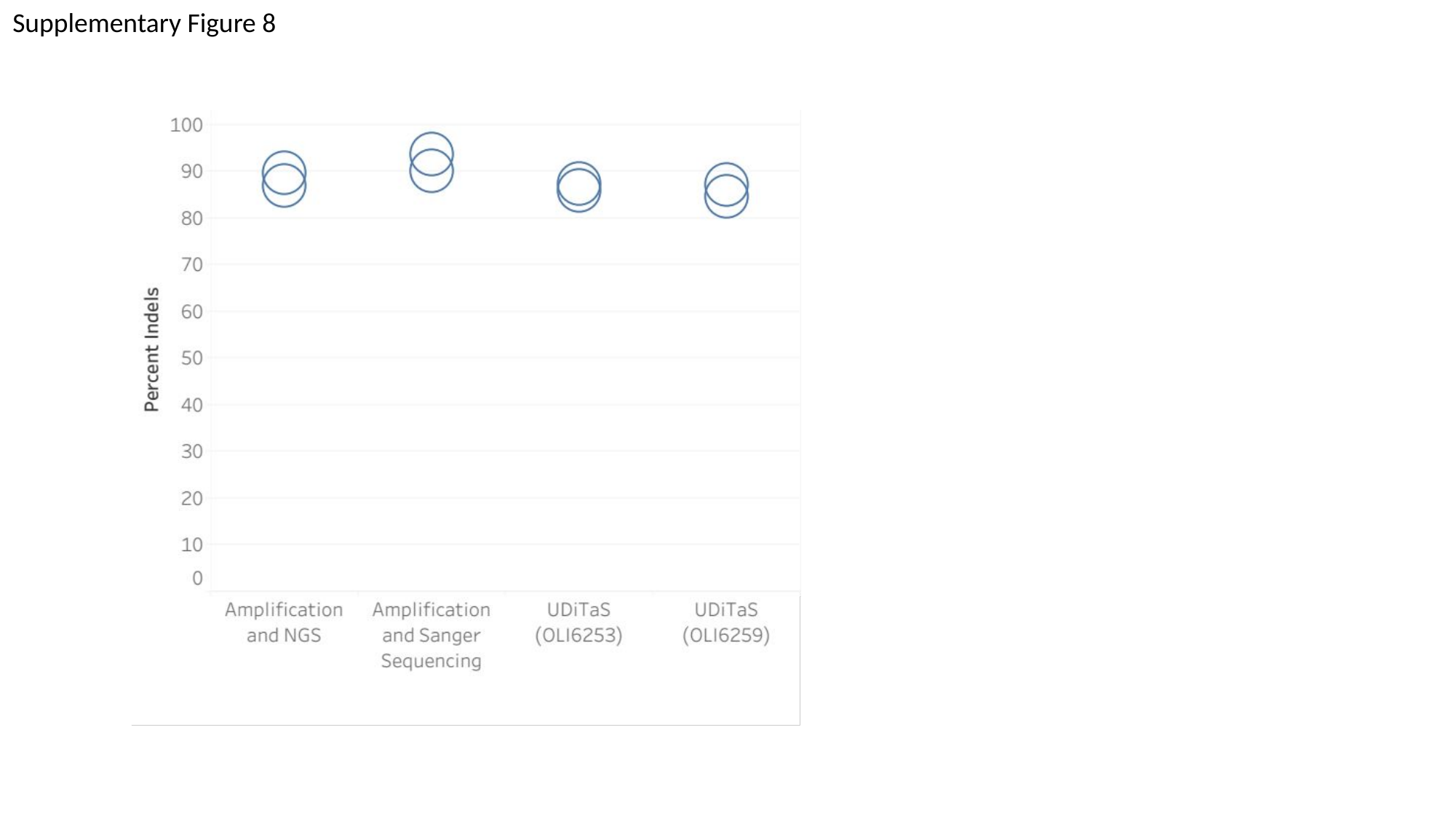

Supplementary Figure 8

Supplement: Supplementary file 9 — Figure S8. Comparison of Indel rates between UDiTaS and other methods. T-Cells edited with the TRAC + B2M guides were analyzed for indel editing at the TRAC locus using PCR-amplification followed by Sanger Sequencing or NGS, in addition to UDiTaS with two different anchor primers. Indel rates were very similar between the methods. (PPTX 81 kb) [file 12864_2018_4561_MOESM9_ESM.pptx]
