# Supplementary material for: UDiTaS™, a genome editing detection method for indels and genome rearrangements
Source: BMC Genomics. 2018 Mar 21;19:212. doi: 10.1186/s12864-018-4561-9 (PMC5861650; doi:10.1186/s12864-018-4561-9)
Supplement: Supplementary file 7 — Figure S6. UDiTaS characterization and comparison to AMP-Seq with plasmid standards. Plasmids containing the CEP290 structural variants a. or the TRAC-B2M balanced translocation b. and c. were synthesized and contain engineered unique SNPs in the insert to identify the plasmid after sequencing. The plasmids were diluted at various levels into mouse genomic DNA and processed through UDiTaS and AMP-Seq using primers for CEP290 a., B2M b. and TRAC c. The number of input plasmids versus the number of plasmids detected is plotted for both UDiTaS and AMP-Seq. Linear regression models and 95% confidence model predictions are displayed on the plots. The parameter β determines the linearity of the method, with values close to 1 indicating more linearity. We used ANOVA p-values to examine differences in β for UDiTaS and AMP-Seq. Below each plot, the table displays the total number of fastq reads sequenced in the reaction, the number of reads mapped to the wild-type amplicon (the most abundant one) and the final number of UMIs counted, for both UDiTaS and AMP-Seq. At all tested loci, UDiTaS shows greater linearity and number of UMIs detected when compared to AMP-Seq. (PPTX 12722 kb) [file 12864_2018_4561_MOESM7_ESM.pptx]

## Slide 1
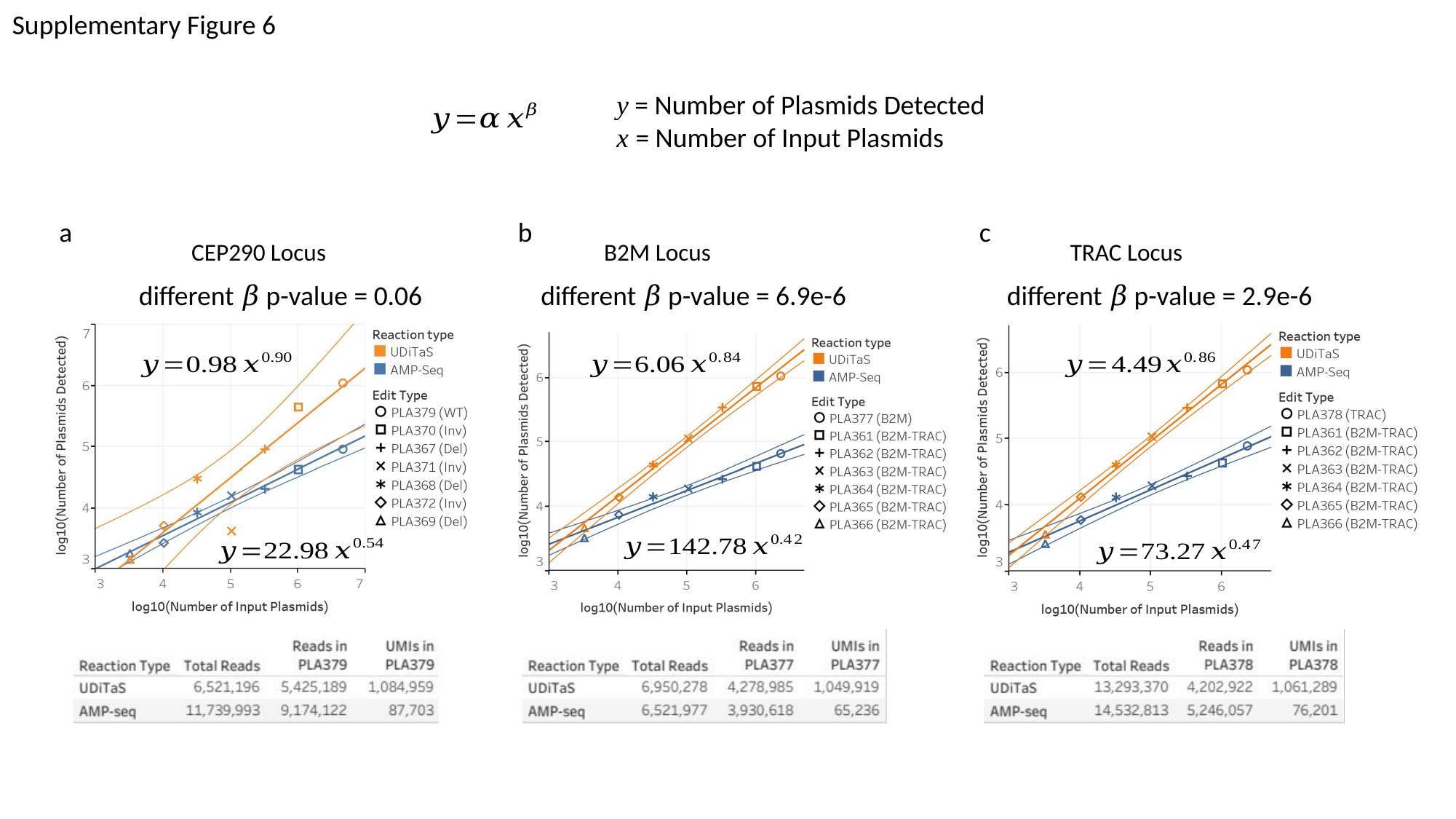

Supplementary Figure 6
y = Number of Plasmids Detected
x = Number of Input Plasmids
a
b
c
CEP290 Locus
B2M Locus
TRAC Locus
different 𝛽 p-value = 0.06
different 𝛽 p-value = 6.9e-6
different 𝛽 p-value = 2.9e-6
